# Utility of the Autism Diagnostic Observation Schedule and the Brief Observation of Social and Communication Change for Measuring Outcomes for a Parent‐Mediated Early Autism Intervention

**DOI:** 10.1002/aur.2449

**Published:** 2020-12-04

**Authors:** Sophie Carruthers, Tony Charman, Nicole El Hawi, Young Ah Kim, Rachel Randle, Catherine Lord, Andrew Pickles, the PACT Consortium

**Affiliations:** ^1^ Department of Psychology Institute of Psychology, Psychiatry and Neuroscience, King's College London London UK; ^2^ National & Specialist Services, Child & Adolescent Mental Health Services Directorate South London and Maudsley NHS Foundation Trust London UK; ^3^ Department of Psychiatry University of California Los Angeles California USA; ^4^ Department of Biostatistics and Health Informatics Institute of Psychology, Psychiatry and Neuroscience, King's College London London UK

**Keywords:** Brief Observation of Social Communication Change, Autism Diagnostic Observation Schedule, autism spectrum disorder, trials, outcome measures, intervention

## Abstract

Measuring outcomes for autistic children following social communication interventions is an ongoing challenge given the heterogeneous changes, which can be subtle. We tested and compared the overall and item‐level intervention effects of the Brief Observation of Social Communication Change (BOSCC), Autism Diagnostic Observation Schedule (ADOS‐2) algorithm, and ADOS‐2 Calibrated Severity Scores (CSS) with autistic children aged 2–5 years from the Preschool Autism Communication Trial (PACT). The BOSCC was applied to Module 1 ADOS assessments (ADOS‐BOSCC). Among the 117 children using single or no words (Module 1), the ADOS‐BOSCC, ADOS algorithm, and ADOS CSS each detected small non‐significant intervention effects. However, on the ADOS algorithm, there was a medium significant intervention effect for children with “few to no words” at baseline, while children with “some words” showed little intervention effect. For the full PACT sample (including ADOS Module 2, total *n*=152), ADOS metrics evidenced significant small (CSS) and medium (algorithm) overall intervention effects. None of the Module 1 item‐level intervention effects reached significance, with largest changes observed for Gesture (ADOS‐BOSCC and ADOS), Facial Expressions (ADOS), and Intonation (ADOS). Significant ADOS Module 2 item‐level effects were observed for Mannerisms and Repetitive Interests and Stereotyped Behaviors. Despite strong psychometric properties, the ADOS‐BOSCC was not more sensitive to behavioral changes than the ADOS among Module 1 children. Our results suggest the ADOS can be a sensitive outcome measure. Item‐level intervention effect plots have the potential to indicate intervention “signatures of change,” a concept that may be useful in future trials and systematic reviews.

**Lay Summary:**

This study compares two outcome measures in a parent‐mediated therapy. Neither was clearly better or worse than the other; however, the Autism Diagnostic Observation Schedule produced somewhat clearer evidence than the Brief Observation of Social Communication Change of improvement among children who had use of “few to no” words at the start. We explore which particular behaviors are associated with greater improvement. These findings can inform researchers when they consider how best to explore the impact of their intervention.

## Introduction

The two core diagnostic domains of autism include difficulties with reciprocal social communication, together with the presence of rigid and repetitive behaviors and interests, and sensory aversions or interests [American Psychiatric Association, 2013]. Goals for many autism interventions, in particular those for young children, include improving social and communication skills, and managing restricted and repetitive behaviors (RRB) that cause challenges [e.g., Grahame et al., 2015; Kasari, Freeman, & Paparella, 2006]. However, although some randomized controlled trials (RCTs) have demonstrated changes in developmental language and play skills [Dawson et al., 2010; Kasari, Paparella, Freeman, & Jahromi, 2008; Rogers et al., 2019], very few have evidenced improvement in the core autism characteristics of reciprocal social communication and RRB [French & Kennedy, 2018; Pickles et al., 2016; Sandbank et al., 2020].

One factor considered to underpin this limited evidence for change in core characteristics is the inadequacy of currently available outcome measures [Anagnostou et al., 2015; Bolte & Diehl, 2013; Green & Garg, 2018; Grzadzinski, Janvier, & Kim, 2020; McConachie et al., 2015; Provenzani et al., 2019; Scahill et al., 2015]. Frequently emphasized is the lack of “sensitivity” to change [Provenzani et al., 2019]. That is, current tools are perhaps not able to discriminate between children making no change at all, and those making small incremental improvements, which may have meaningful implications for daily life or important downstream effects.

One commonly used tool is the Autism Diagnostic Observation Schedule [Lord et al., 2012], the “gold‐standard” diagnostic tool, often used to characterize the sample and, in many studies, to track outcomes [Cunningham, 2012]. Trained administrators use a series of semi‐structured tasks to elicit communication and social interaction for approximately 45–60 min, using one of six modules matched to the individual's language and developmental level [including Bal et al., 2020]. Designed to inform diagnosis, its properties reflect the aim to classify children with and without autism, a division considered to be relatively stable. To overcome the variation in scores across modules, a mapping of ADOS module total to Calibrated Severity Scores (CSS) has been proposed [Gotham, Pickles, & Lord, 2009]. However, the limited three‐ or four‐point ADOS item scoring range may have the potential to mask intermediate improvements, and this may be exacerbated by the further reduction to the 10‐point CSS. Though a few trials have reported significant intervention effects with the ADOS social communication algorithm score [Aldred, Green, & Adams, 2004], the CSS [Pickles et al., 2016] or the diagnostic classification [Solomon, Van Egeren, Mahoney, Huber, & Zimmerman, 2014], the majority do not find a significant difference between groups [Dawson et al., 2010; Fletcher‐Watson et al., 2016; Oosterling, Visser, et al., 2010; Rogers et al., 2012; Rogers et al., 2019; Wetherby et al., 2014]. Several reviews have questioned the appropriateness of the ADOS as an outcome measure [Anagnostou et al., 2015; Cunningham, 2012; McConachie et al., 2015]. One factor that may play a role in the lack of significant results is intervention length, where the ADOS is less likely to capture change over shorter durations [e.g., Fletcher‐Watson et al., 2016].

In response to criticisms of outcome measures, Grzadzinski et al. [2016] developed the Brief Observation of Social and Communication Change (BOSCC), intended to offer a more efficient alternative to a repeat ADOS at trial endpoint. Social communication and RRB subdomains are scored from a 10–12 min adult‐child interaction across items with a six‐point scale, a larger range than that of the ADOS items. The standard BOSCC uses naturalistic adult‐child interactions, while an adapted version, named the ADOS‐BOSCC, can be coded from sections of video‐recorded ADOS assessments [Kim, Grzadzinski, Martinez, & Lord, 2019]. The ADOS‐BOSCC can therefore be used to evaluate intervention efficacy using retrospective data from completed studies that have videotapes of ADOS administrations. The two versions may have different merits. While the naturalistic BOSCC can be used flexibly to score a child's interaction with whomever is appropriate for that intervention, the ADOS‐BOSCC may be more sensitive to RRB behaviors where the more structured ADOS tasks can elicit them [Grzadzinski et al., 2020]. However, some caution is needed when using the RRB subdomain as the behaviors can be harder to score reliably or skewed as a result of their infrequent nature [Grzadzinski & Lord, 2018].

Preliminary findings from four studies analyzing the Module 1 BOSCC or ADOS‐BOSCC with samples of children under 6 years with minimal verbal language before and after an intervention suggest the measures have strong psychometric properties [Grzadzinski et al., 2016; Kim et al., 2019; Kitzerow, Teufel, Wilker, & Freitag, 2016; Pijl et al., 2018]. The studies by Grzadzinski et al. [2016] and Kim et al. [2019] studied change over 6 and 9 months, respectively, while the other two studies studied change over a longer period of 12 and 15 months [Kitzerow et al., 2016; Pijl et al., 2018]. High inter‐rater and test–retest reliability and appropriate indicators of convergent validity and discriminant validity were evidenced. With regards to sensitivity to change, all four studies reported significant reductions (improvements) in the BOSCC or ADOS‐BOSCC total score with small‐moderate effect sizes (ES). For social communication, significant moderate improvements were reported only by the two studies reporting on the ADOS‐BOSCC [Kim et al., 2019; Kitzerow et al., 2016], while Pijl et al. [2018], using the standard BOSCC, was the only study to report significant change for RRB. In contrast to the consistent significant reductions in BOSCC or ADOS‐BOSCC total score, only one of the four studies reported a significant improvement in the concurrently obtained ADOS CSS [Pijl et al., 2018]. The absence of control groups and a randomized design in these four studies prevents any inference that the improvements were related to the interventions. Three moderate‐size RCTs applying the standard naturalistic BOSCC as an outcome measure reported small and not significant ES [Divan et al., 2019; Fletcher‐Watson et al., 2016; Nordahl‐Hansen, Fletcher‐Watson, McConachie, & Kaale, 2016]. Applying the standard BOSCC coding scheme to a non‐standard, structured parent–child interaction, one study found a large significant intervention effect [Gengoux et al., 2019]. Existing results on the BOSCC and ADOS‐BOSCC are therefore inconsistent, with no RCT yet having used the ADOS‐BOSCC.

Change in autistic characteristics is often only described at the total or subdomain (social communication and RRB) level. However, when considering the impact of an intervention, change within individual behaviors may provide greater insight into underlying patterns of effect [e.g. Rose, Trembath, Keen, & Paynter, 2016]. Item‐level “treatment effect” profiles could reveal “signatures of change,” indicating which behaviors are associated with relatively greater or lesser change following a specific intervention approach. Especially as part of systematic reviews, these profiles could facilitate understanding of which interventions are optimal for different goals, be informative for hypotheses of intervention mechanism, and identify weaker areas of effect to address.

It is challenging to determine the extent to which the lack of evidenced improvement in core autism characteristics is due to unresponsive outcome tools, and/or limited effectiveness of interventions [Grzadzinski et al., 2020]. The Preschool Autism Communication Trial (PACT) [Green et al., 2010] was a large randomized controlled trial of a parent‐mediated social and communication therapy for young children with autism. Though the original publication demonstrated that PACT was associated with small nonsignificant effects on the ADOS Social Communication scale alone, a subsequent analysis [Pickles et al., 2016] used the CSS (including both social communication and RRB) for which a significant intervention effect at endpoint with a log proportional odds ratio of 0.64 was found. With an evidenced intervention effect, the PACT trial data provide a good opportunity for the ADOS‐BOSCC to be tested and compared with the ADOS‐2 algorithm and CSS, and for the profile of intervention effects on both instruments to be explored at an item‐level.

This study therefore aimed to:

1. Test the psychometric properties of the ADOS‐BOSCC and, where informative, the ADOS algorithm and ADOS CSS.
2. Test and, where possible, compare sensitivity to change and intervention effect sizes of the ADOS‐BOSCC, ADOS‐2 algorithm, and ADOS CSS.
3. Explore whether the ADOS‐BOSCC and ADOS can inform us about the item‐level intervention “signature of change” for PACT.

## Methods

### *Participants and Study Design*

The PACT trial was conducted in London, Manchester, and Newcastle, UK, with 152 families with a child aged 2 years to 4 years and 11 months who met criteria for core autism, of whom 146 (95%) were retained to 13‐month outcome. One hundred and seventeen children received a Module 1 ADOS (see Table 1) and 35 received a Module 2 ADOS (see Table S1). Ethical approval was given by the Central Manchester Multicentre Research Ethics Committee (05/Q1407/311). Exclusion criteria, study design, and sample characteristics are reported in Appendix S1.

**Table 1:** Baseline Characteristics of Module 1 Children by Intervention Group

	PACT (*n* = 60)	TAU (*n* = 57)
Child age (months; mean, range)	44 (26–60)	44 (24–60)
Girl, *n* (%)	4 (7)	6 (11)
Parents' ethnic origin, *n* (%)		
Both white	34 (57)	28 (49)
Mixed^a^	2 (3)	8 (14)
Non‐white	24 (40)	21 (37)
Education (one parent with qualifications after age 16y), *n* (%)	51 (85)	40 (63)
Socioeconomic status^b^, *n* (%)	39 (65)	34 (60)
MSEL non‐verbal age equivalent (months; mean, SD)	22.8 (5.9)	21.6 (5.6)

*Note*. Data are number (%), unless otherwise indicated.

MSEL: Mullen Scales of Early Learning; PACT: Preschool Autism Communication Trial; TAU: treatment‐as‐usual.

^a^ One white parent and the other non‐white.

^b^ Dichotomised as at least one parent in professional or administrative occupation *versus* all others.

### *PACT* *Intervention*

The PACT intervention targeted social interactive and communication skills in autism. The rationale was that children with autism would respond with enhanced communicative and social development to a style of parent communication adapted to their impairments. The intervention consisted of one‐to‐one clinic sessions between therapist and parent with the child present. After an initial orientation meeting, families attended biweekly 2 h clinic sessions for 6 months followed by monthly booster sessions for 6 months (total 18). Between sessions, families were also asked to do 30 min of daily home practice. Details of the intervention are reported in Green et al. [2010].

### *Outcome Measures*

#### *ADOS*

Research‐reliable researchers administered and scored the ADOS‐G [Lord et al., 2000] for all children at baseline and endpoint. In the original trial, the same module was administered at baseline and endpoint to facilitate tracking of change as the CSS was not yet available. Researchers scoring the assessments were blind to group but not timepoint. The original ADOS‐G raw scores were used to calculate the standardized ADOS‐2 algorithm scores [Lord et al., 2012] and ADOS CSS [Gotham et al., 2009; Hus, Gotham, & Lord, 2014]. Four items within the Module 1 total differ for children who use “few to no words” or “some words” (rates of use in Appendix S1). The algorithm total score is then converted into the CSS (range 1–10, where higher represents a greater level of autistic characteristics) according to the language level and chronological age of the child. Inter‐rater reliability from 66 ratings across 15 videos (calculated through structural equation models [SEM] with a maximum likelihood missing values estimator) was good for the total ADOS‐2 score (0.84 [95% CI 0.72, 0.95]), good for the Social Affect subdomain (0.79 [CI 0.65, 0.93]) and moderate for the RRB subdomain (0.53 [CI 0.29, 0.77]). Inter‐rater reliability could not be calculated for the CSS as the database for the ADOS algorithm reliability was composed at the time of the PACT trial before the CSS was published and did not include details on the child age.

#### *ADOS‐BOSCC*

The ADOS‐BOSCC (Version July 27, 2017) [Kim et al., 2019] provides an adapted BOSCC coding system for scoring behavior observed during ADOS assessments. Consisting of the standard 15 items plus an additional item for Requesting Behaviors, item scores range from 0 (autistic characteristic is not present) to 5 (autistic characteristic is present). Thirteen core items (maximum score 65) consist of nine items for a Social Communication subdomain (maximum score 45) and four items for an RRB subdomain (maximum score 20). An additional three items measure activity level, irritability and anxiety, for which we report reliability but are not used in other analyzes. The ADOS‐BOSCC is coded from 12 min of videotaped ADOS assessments. Segment A includes 3 min each of Free Play and Bubble Play and segment B includes 3 min each of Birthday Party and Anticipation of Routine with Objects. If either segment is under 6 min, up to 3 min of Response to Joint Attention for Segment A or Snack for Segment B is coded. Only the Module 1 ADOS‐BOSCC was available at the time of analysis and therefore only those children who were administered a Module 1 ADOS were included in the ADOS‐BOSCC analysis.

Four ADOS‐BOSCC trained coders, blind to timepoint and group, coded the videos. Forty‐eight ratings from 12 videos were used to calculate ICCs (two way, mixed) for inter‐rater reliability from averaged sum scores of the videos. These were good [Koo & Li, 2016], being 0.89, 95% CI (0.74, 0.96) for the total score, 0.89 (0.74, 0.97) Social Communication subdomain and 0.73 (0.50, 0.90) for RRB. Individual item ICCs ranged between 0.46 and 0.93 (Table S3). Two items (Eye Contact and Mannerisms) had poor reliability and fell below 0.50. Further details, along with details of measures used as covariates or correlates, are reported in Appendix S1 including Table S2.

### *Data Analysis*

We had 104 complete pairings of baseline and endpoint Module 1 data points for both ADOS‐BOSCC and ADOS, which were used in all analyzes in which the two measures are compared.

Item‐rest correlations were reported to explore within‐subscale consistency for the 13 core ADOS‐BOSCC items using baseline data, where a recommended range is between 0.2 and 0.7 [Streiner, Norman, & Cairney, 2015]. To assess the fit of the two ADOS‐BOSCC subdomains in this sample, factor analysis was conducted in MPlus 8 using a geomin oblique rotation, with items 1–9 representing Social Communication and items 10–13 representing RRB [Grzadzinski et al., 2016; Kim et al., 2019]. All items were included from both segments totaling 26 items, each treated as categorical. Baseline and endpoint data were included as two records per child, with the complex survey adjustment for clustered data used to account for the non‐independence of observations from the same child. Goodness of fit was evaluated with RMSEA and CFI, where satisfactory fit is indicated by values below 0.08 and above 0.90, respectively [Kline, 2015; MacCallum, Browne, & Sugawara, 1996]. Extensive psychometric analyzes on the ADOS have previously been conducted including several replications of the two‐factor factor analysis [Gotham et al., 2008; Gotham, Risi, Pickles, & Lord, 2007; Oosterling, Roos, et al., 2010].

Correlations with baseline and change scores were conducted between the three metrics, the Mullen Scales of Early Learning (MSEL) [Mullen, 1995] non‐verbal age‐equivalent and Vineland Adaptive Behavior Scales Expressive and Receptive Language age‐equivalent scores [Sparrow, Cicchetti, & Balla, 2006] to determine convergent validity. Pre‐post correlations were also conducted for each outcome measure. Correlations are interpreted using *r* of ≥0.1 represents a small ES, ≥0.3 a medium ES and ≥0.5 a large ES [Cohen, 1988]. Spearman correlations (*r*_s_) were used for skewed variables.

We examined evidence of sensitivity to change using paired t‐tests. Where Cohen's *d* ES are reported, they are interpreted as ≥0.2 is a small effect, ≥0.5 a medium effect, and ≥0.8 a large effect [Cohen, 1988].

In a randomized trial setting, analysis of covariance (ANCOVA) estimates the same parameter as analysis of change scores, but generally does so with greater efficiency on account of exploiting the pre‐post correlation in a context where randomization assures regression to a common mean can be assumed. We used a structural equation model setup equivalent to traditional ANCOVA to exploit the desirable missing data properties of full maximum likelihood (traditional ANCOVA results are also reported in Table S9). In light of the difference in mapping of ADOS scores to CSS for verbal and non‐verbal Module 1 children, the ADOS analysis was stratified by baseline level of language, and additionally by Module for analyzes including Module 2 children. An ES pooled over strata was calculated based on the standard deviation of the measure at baseline for each stratum, weighting the stratum specific estimates by their precision. Alternative ES using standard deviation of change were also calculated. Covariates were the same as those used in the original trial analysis: centre, age group (> or ≤ 42 months), sex, verbal ability (expressive raw score on the Preschool Language Scales) [Zimmerman, Steiner, Pond, Boucher, & Lewis, 1997], non‐verbal ability (MSEL), parental educational qualifications, and socioeconomic status. Overall Module 1 ES estimates for ADOS‐BOSCC, ADOS algorithm, and ADOS CSS were tested with bootstrapping. Intervention effect models were estimated for ADOS‐BOSCC, ADOS algorithm, ADOS CSS total, subdomains, and the items within the ADOS‐BOSCC and ADOS algorithm. Results are presented in forest plots. All confidence intervals are 95% with those for the item‐level intervention effects adjusted using the Dubey/Armitage‐Parmar method [for simulations and explanation, see Vickerstaff, Omar, & Ambler, 2019] to account for there being multiple correlated items. The ADOS‐BOSCC total score intervention analysis was preregistered at osf.io/a93t8. All other analyzes should be considered exploratory and changes to the pre‐registered analysis are described in Appendix S1.

## Results

### *Psychometric Properties*

#### *ADOS‐BOSCC* *item‐rest correlations and factor analysis*

The majority of item‐rest correlations were within the recommended range of 0.2–0.7 (Table S3). Two items, Integration and Requesting, had item‐rest correlations above 0.7. One item, Mannerisms, had an item‐rest correlation below 0.2.

Consistent with previous studies [Grzadzinski et al., 2016; Kim et al., 2019], the two‐factor solution fitted relatively well (RMSEA = 0.074, CFI = 0.923). Item loadings are reported in Table S4. All items under the Social Communication factor had loadings above 0.40 (range 0.41–0.88). For the RRB factor, “Play” from both segments and “Repetitive/Stereotyped Behavior” from segment A had loadings above 0.40 (range 0.42–0.80). Other RRB items fell below 0.40.

#### *Pre‐Post* *correlations*

Correlations between baseline and endpoint scores were highly correlated for the ADOS‐BOSCC (*r* = 0.66, *P* < 0.001) and for the ADOS algorithm (*r* = 0.52, *P* < 0.001) and moderately correlated for the ADOS CSS (*r*_*s*_ = 0.34, *P* < 0.001).

#### *Convergent validity*

Within the respective ADOS and ADOS‐BOSCC total scores, correlations for baseline and for change scores were moderate‐high (Table S5). Correlations between the metric subdomains are reported in Table S6.

At baseline, small‐moderate negative correlations were found for nonverbal IQ with all three metrics (Table S5). For the language measures, there were small‐moderate negative baseline correlations with the ADOS‐BOSCC and ADOS algorithm, small‐moderate positive correlations for their respective change scores, and no significant correlations with ADOS CSS.

### *Module 1:* *ADOS‐BOSCC,* *ADOS* *Algorithm, and* *ADOS CSS*

#### *Sensitivity to change*

All three metrics, the ADOS‐BOSCC, ADOS algorithm, and ADOS CSS, had significant pre‐post change scores for PACT and TAU, indicating improvement in the total scores across the sample (Table 2). For social communication, the ADOS‐BOSCC and ADOS algorithm detected significant improvements for both groups, while the ADOS CSS only found significant improvements for the PACT group. No measure of RRBs detected significant reduction for either group. ES for pre‐post mean differences were broadly similar across metrics for most domains.

**Table 2:** Pre‐Post Change Scores for ADOS‐BOSCC and ADOS Module 1 by Intervention Group

	Baseline		Endpoint		TAU change		PACT change	
	Mean (SD)		Mean (SD)		Mean difference (SD)	Effect size (*d*_*z*_)	Mean difference (SD)	Effect size (*d*_*z*_)
	TAU	PACT	TAU	PACT				
ADOS‐BOSCC Total	37.7 (9.24)	37.4 (8.66)	33.8 (11.1)	31.4 (11.2)	−3.93 (8.52)*	0.46	−6.02 (8.44)**	0.71
ADOS Total	20.9 (2.99)	21.1 (3.97)	19.5 (5.01)	17.7 (5.63)	−1.42 (4.26)*	0.33	−3.42 (4.84)**	0.71
ADOS CSS	7.94 (1.38)	8.04 (1.44)	7.40 (1.68)	6.96 (1.78)	−0.54 (1.88)*	0.29	−1.08 (1.72)**	0.62
ADOS‐BOSCC SC	28.8 (7.08)	28.8 (6.91)	25.4 (8.71)	23.6 (8.92)	−3.39 (6.66)**	0.51	−5.25 (7.46)**	0.70
ADOS SA algorithm	16.1 (2.38)	15.7 (2.98)	14.7 (4.23)	13.2 (4.72)	−1.48 (3.53)*	0.42	−2.77 (4.50)**	0.62
ADOS CSS SA	7.73 (1.39)	7.75 (1.47)	7.21 (1.95)	6.63 (1.86)	−0.52 (2.12)	0.25	−1.12 (2.05)**	0.54
ADOS‐BOSCC RRB	8.85 (3.73)	8.53 (3.32)	8.31 (3.58)	7.76 (3.56)	−0.54 (4.23)	0.13	−0.77 (3.34)	0.23
ADOS RRB algorithm	4.77 (1.64)	5.12 (1.97)	4.83 (1.85)	4.46 (1.79)	−0.06 (2.17)	−0.03	−0.65 (1.96)	0.33
ADOS CSS RRB	8.04 (1.51)	8.33 (1.48)	8.04 (1.62)	7.77 (1.81)	0.00 (2.02)	0.00	−0.56 (1.86)	0.30

*Note*. A negative change score indicates improvement on the scale. Paired sample *t*‐tests tested within‐subdomain change. *n*=104 [TAU n = 52; PACT n = 52].

ADOS: Autism Diagnostic Observation Schedule; ADOS‐BOSCC: Brief Observation for Social Communication Change–version for ADOS; *d*_*z*_: Cohen's *d*_*z*_ effect size for correlated samples; PACT, Preschool Autism Communication Trial; SA: social affect; SC: social communication; RRB: restricted and repetitive behaviors; TAU: treatment‐as‐usual.

*P < 0.05; **P < 0.01.

#### *Intervention effects*

**Figure 1:**
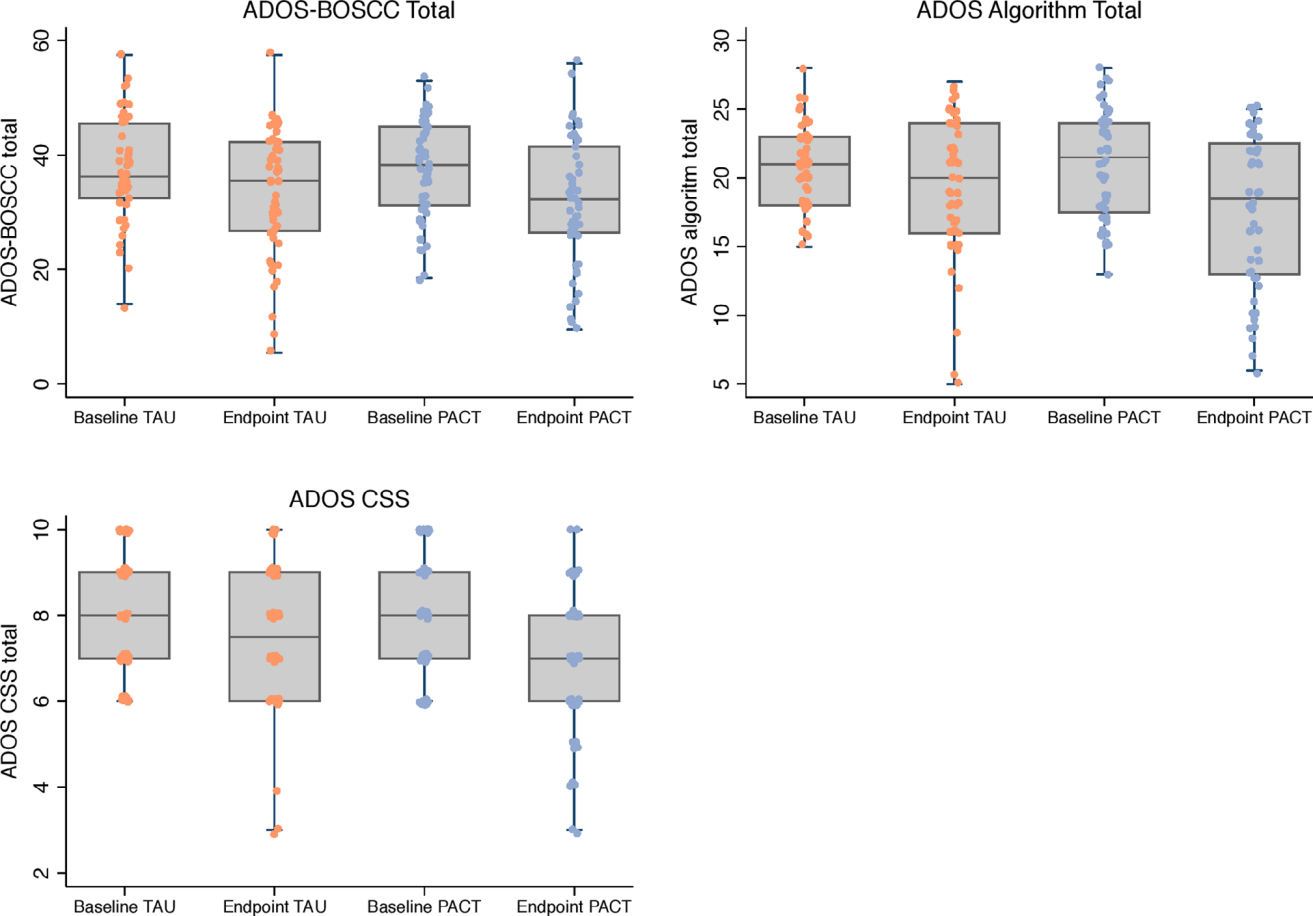
Box plots with scatter of ADOS‐BOSCC, ADOS algorithm, and ADOS CSS totals at baseline and endpoint across intervention groups for Module 1. ADOS: Autism Diagnostic Observation Schedule; ADOS‐BOSCC: Brief Observation of Social and Communication Change‐version for ADOS; PACT: Preschool Autism Communication Trial; TAU: treatment‐as‐usual.

**Figure 2:**
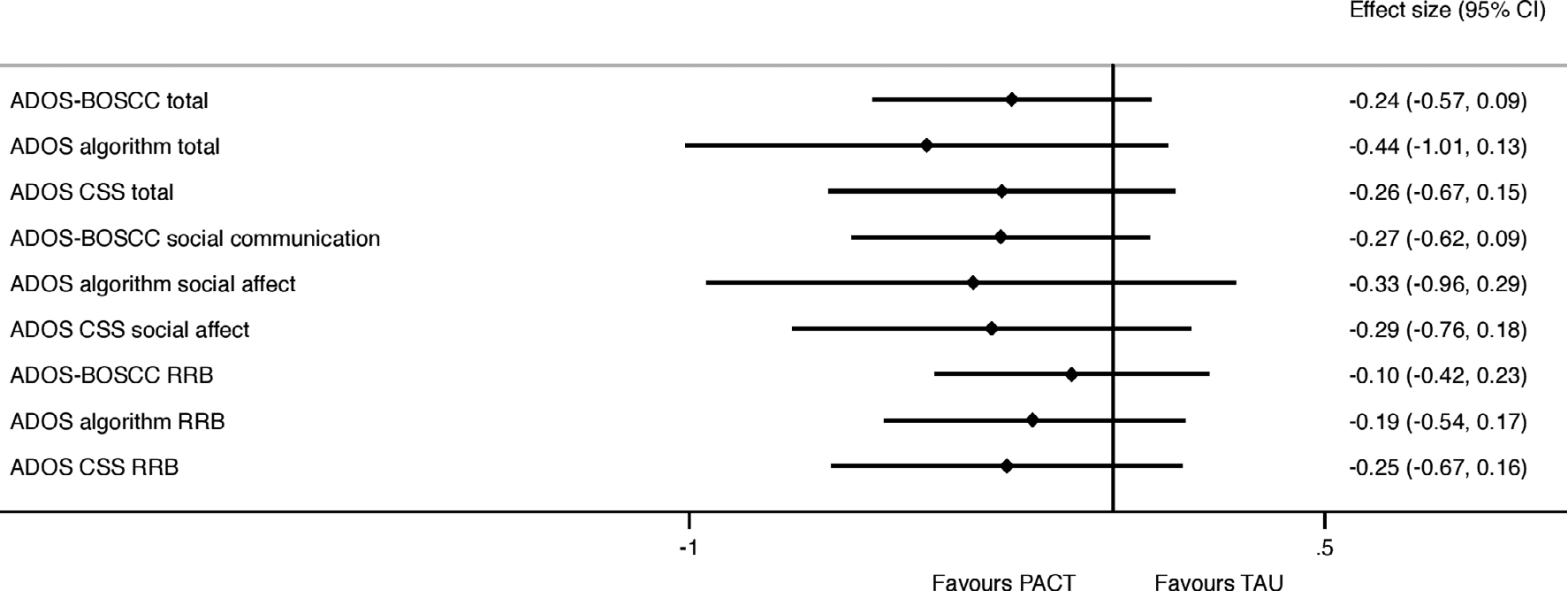
Forest plot of intervention effect size estimates for the Module 1 total and subdomain scores. Negative effect sizes are in favor of PACT. ADOS: Autism Diagnostic Observation Schedule; ADOS‐BOSCC: Brief Observation of Social and Communication Change‐version for ADOS; CSS: Calibrated Severity Scores; PACT: Preschool Autism Communication Trial; RRB: restricted and repetitive behaviors; TAU: treatment‐as‐usual.

Scatter box plots by intervention group for baseline and endpoint ADOS‐BOSCC, ADOS algorithm, and ADOS CSS totals are shown in Figure 1 and for SA and RRB subtotals in Figures S1 and S2. The ADOS‐BOSCC total and the ADOS CSS detected a non‐significant intervention effect, with small ES of −0.24 (95% CI −0.53, 0.17) and −0.26 (95% CI −0.67, 0.15), respectively (Table 3, Fig. 2). The ADOS algorithm overall total also detected a non‐significant intervention effect for Module 1, though with a larger point ES estimate of −0.44 (95% CI ‐1.01, 0.13). Pairwise tests revealed the differences between the three ES are not significant (Table S8). Within the two ADOS strata, a large significant intervention effect was found for children who were in the “few to no words” category at baseline with an ES of −0.73 (95% CI −1.43, −0.02). In contrast, children in the “some words” category at baseline were associated with a non‐significant intervention effect with negligible ES of 0.09 (95% CI ‐0.87, 1.05). A Wald test revealed these ES were not significantly different. As presented in Table S7, this differential pattern of effect reflects children with “few to no words” benefiting from PACT more than TAU, where improvement is minimal, whereas children with “some words” benefit equally from PACT and TAU. For comparison with the SEM output, the ANCOVA results are reported in Table S9.

**Table 3:** Intervention Effect Results for ADOS‐BOSCC, ADOS Algorithm, and ADOS CSS for Module 1

	ADOS‐BOSCC		ADOS algorithm				ADOS CSS	
Strata			Few to no words		Some words			
	Coefficient	95% CI	Coefficient	95% CI	Coefficient	95% CI	Coefficient	95% CI
PACT intervention	−2.13	−5.07, 0.81	−2.18*	−4.30, −0.07	0.25	−2.41, 2.92	−0.37	−0.94, 0.21
Covariates								
Nonverbal IQ	−0.72**	−1.07, −0.37	−0.33*	−0.64, −0.03	−0.39*	−0.62, −0.16	−0.13**	−0.19, −0.06
Expressive language	−0.95**	−1.39, −0.52	−0.36	−0.77, 0.05	0.31	−0.15, 0.78	0.01	−0.07, 0.09
London vs. Manchester	−1.69	−5.31, 1.94	−4.07**	−6.36, −1.79	−0.19	−3.90, 3.52	−0.96*	−1.64, −0.28
London vs. Newcastle	−1.03	−5.06, 3.01	−1.83	−4.58, 0.91	−0.51	−3.95, 2.92	−0.72	−1.47, 0.04
Sex	1.84	−3.29, 6.96	1.26	−2.13, 4.65	0.47	−4.15, 5.08	0.54	−0.42. 1.50
Age group	1.68	−1.55, 4.90	1.58	−0.43, 3.60	−0.53	−3.56, 2.49	−0.08	−0.69, 0.52
Parent's job	1.72	−1.74, 5.18	1.06	−1.07, 3.20	−1.82	−5.56, 1.93	0.28	−0.37, 0.93
Parent's education	0.73	−3.12, 4.58	1.00	−1.50, 3.50	4.34	0.05, 8.63	0.25	−0.47, 0.97
Effect size (95% CI)	−0.24 (−0.57, 0.09)		−0.73* (−1.43, −0.02)		0.09 (−0.87, 1.05)		−0.26 (−0.67, 0.15)	
	−0.44 (−1.01, 0.13)						

*Note*. Negative effect sizes are in favor of PACT. Effect sizes are calculated using the pooled standard deviation at baseline.

ADOS: Autism Diagnostic Observation Schedule; ADOS‐BOSCC: Brief Observation of Social Communication Change‐version for ADOS; CSS: Calibrated Severity Scores.

*P < 0.05; **P < 0.001.

For Social Communication or Social Affect subdomains, the ADOS‐BOSCC, ADOS algorithm, and ADOS CSS all identified small non‐significant ES (see Fig. 2 and Table S10‐11). Among the RRB subscales, the ADOS CSS had a small non‐significant effect, while the ADOS algorithm and ADOS‐BOSCC had negligible ES. Across the ADOS algorithm strata, the ES estimates were larger for “few to no words” than “some words” children. Alternative ES using the change score variation are reported in Table S12.

### *Full* *PACT* *Sample:* *ADOS* *Algorithm and* *ADOS CSS*

#### *Sensitivity to change*

For the full sample, the ADOS algorithm and ADOS CSS had significant pre‐post change scores for PACT and TAU, indicating improvement in the total scores and social communication across the sample (Table S13). Both metrics detected significant reduction in RRB behaviors for the PACT group and no change in the TAU group. ES for pre‐post mean differences were broadly similar across metrics for most domains.

#### *Intervention effects*

Combining Module 1 and Module 2 children, a significant and moderate ES of −0.59 (95% CI −0.97, −0.22) was found for the stratified analysis of ADOS algorithm (Table S14) and significant but small ES for each of the SA and RRB domain scores (Fig. S3 and Tables S15 and S16). The ADOS CSS had a smaller but nonetheless significant ES of −0.45 (95% CI −0.75, −0.14), with small effects for SA and RRB, significant only for RRB (Fig. S3 and Table S16). ES using change score variation are presented in Table S12.

### *PACT* *Signature of Change*

#### *Module 1 intervention “signature of change”*

**Figure 3:**
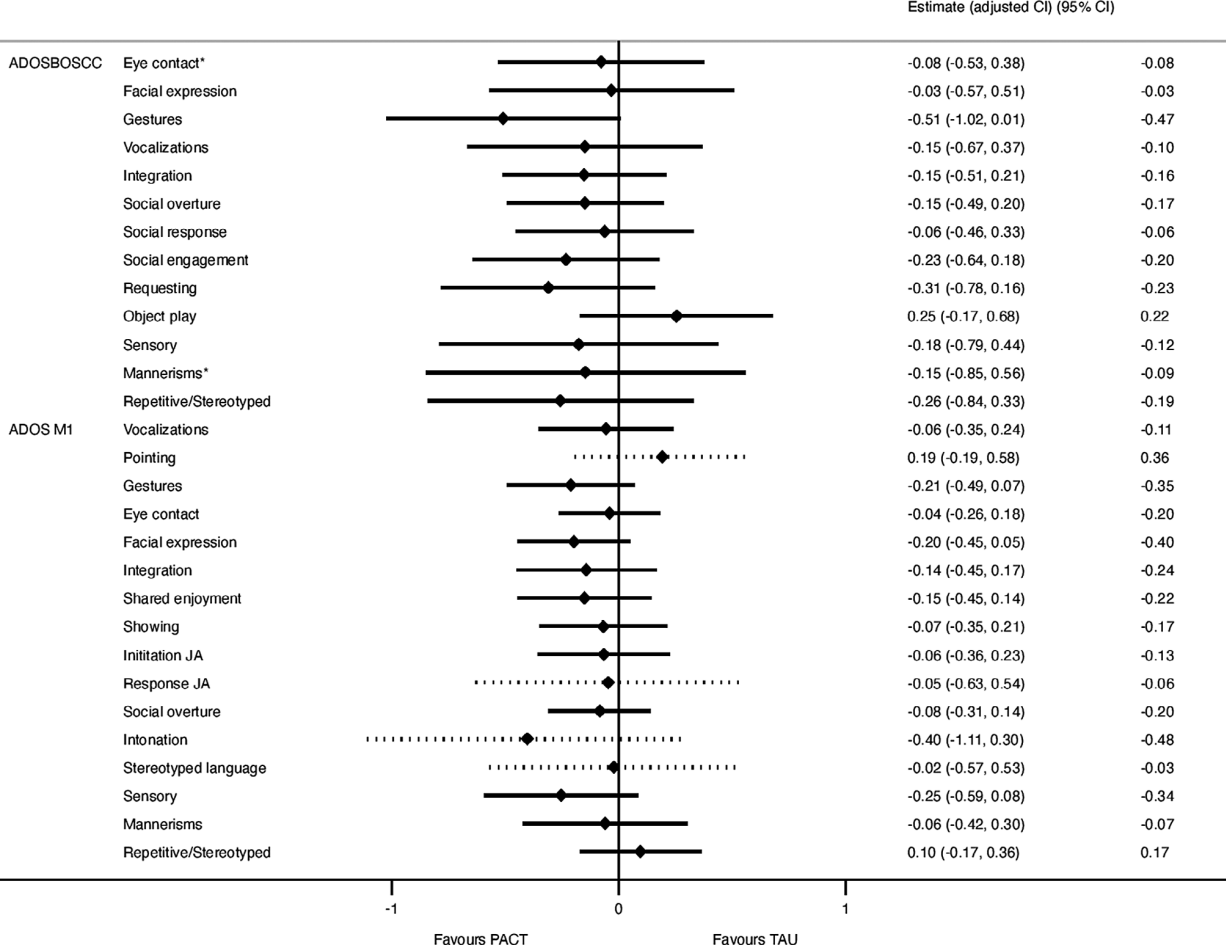
Forest plot of intervention “signature of change”: Model effect estimates for the items of the ADOS‐BOSCC and ADOS (Module 1) with 95% confidence intervals corrected for multiple comparisons within each measure. This graph plots model estimates, while effect sizes are listed in the right‐hand column. Confidence intervals of the model estimates were corrected using the Dubey/Armitage‐Parmar adjustment, which accounts for there being multiple correlated outcomes. Fourteen items make up the ADOS‐2 Module 1 score, but four items differ depending on the language level of the child (indicated with dotted confidence intervals). The intervention models for “Response to Joint Attention” and “Intonation” are therefore only conducted with the 47 Module 1 children who remained in the “Few to No Words” category at both timepoints. The intervention models for “Pointing” and “Stereotyped Language” are only conducted with the 35 children who remained in the “Some Words” category at both time points. *Items marked with an asterisk had poor inter‐rater reliability. ADOS: Autism Diagnostic Observation Schedule; ADOS‐BOSCC: Brief Observation of Social and Communication Change‐version for ADOS; JA: joint attention; M1: Module 1.

At the item‐level (Fig. 3), across ADOS‐BOSCC and ADOS algorithm, no items reached significance. For the ADOS‐BOSCC, Use of Gesture showed the largest improvement with a small ES. On the ADOS, Intonation was the largest improver, but with wide confidence intervals on account of this item applying only to the “some words” stratum. Facial Expression and Use of Gesture were also among the larger improvers, but ES remained small.

#### *Module 2 intervention “signature of change”*

Among ADOS Module 2 item‐level intervention effects (Fig. S4), Mannerisms and Repetitive Interests or Stereotyped Behaviors reached significance, with large effects. Rapport also had a moderate ES but did not reach significance. All other items had small or negligible ES.

## Discussion

This study aimed to explore and compare the ADOS‐BOSCC and the ADOS as outcome measures using the PACT trial. In the original PACT trial, the pre‐specified *modified* ADOS‐G social‐communication scale had shown a non‐significant small to moderate treatment effect [Green et al., 2010]. However, follow‐up work using the ADOS CSS, which spanned SA and RRBs, estimated effects as significant and of moderate size [Pickles et al., 2016]. In this paper, we test and compare the results for ADOS‐BOSCC, a stratified analysis of the ADOS‐2 total algorithm score and the ADOS CSS for the original trial 13‐month endpoint and explore whether item‐level analyzes could inform us about the PACT “signature of change.” Aside from the intervention analysis for the ADOS‐BOSCC, all other analysis should be considered exploratory.

For the Module 1 children, where a three‐way comparison was possible, no measure yielded significant intervention effects, and no measure performed significantly better than any other. Contrary to expectation, the largest ES was obtained with the ADOS algorithm total, with those for the ADOS‐BOSCC and ADOS‐CSS being about half the size, though these differences were not significant. The requirement to stratify the ADOS algorithm analyzes by baseline verbal ability highlighted possible greater intervention effects among those with “few to no words” compared to “some.” This finding should be treated with caution given the small sample size and absence of prior hypothesis.

Using the full PACT sample, in line with Pickles et al. [2016], both the ADOS total algorithm score and ADOS CSS detected a significant medium and small sized intervention effect at intervention endpoint, respectively. Our results evidence how the RRB subdomain, particularly among Module 2 children, is a substantial component of this overall ADOS effect and indicates why the CSS analysis in the Pickles et al. [2016] study revealed a different result to that of the original pre‐specified analysis with only the modified ADOS‐G social communication subdomain [Green et al., 2010].

Among Module 1 children, no item‐level intervention effects reached significance. Significant item‐level effects were observed for Mannerisms and Repetitive Interests and Stereotyped Behaviors for Module 2 children.

### *Psychometric Properties*

Inter‐rater reliability for the ADOS‐BOSCC was high at the total and subdomain levels. As this is the largest BOSCC coding project so far published, this is encouraging for future trials. At the item‐level, eye contact and mannerisms were found to have poor reliability, likely as a result of the challenges of coding these behaviors from low definition videos (as was the case for recordings at the time of the PACT trial), particularly when brief. For the ADOS‐BOSCC, a two‐factor factor structure was supported, in line with results of previous studies [Grzadzinski et al., 2016; Kim et al., 2019]. This study also confirmed the convergent validity of the ADOS‐BOSCC in detecting behavioral changes in line with those measured by parent‐reported language skills (Table S5) [Kim et al., 2019]. Change in the ADOS algorithm total was correlated with parent‐rated changes in expressive, but not receptive, language. In line with the intention that the CSS be independent of changes to verbal IQ, the CSS change score did not correlate with language measures.

### *Module 1* *ADOS‐BOSCC* *and* *ADOS*

#### *Sensitivity to change*

The mean pre‐post change scores for total and subdomains demonstrated broadly similar patterns across the three metrics, indicating that both the ADOS‐BOSCC and ADOS were sensitive to change in autistic characteristics over time (Table 2). This is in contrast to previous studies where the ADOS‐BOSCC, but not the ADOS CSS, demonstrated significant mean pre‐post differences for children who had received intervention [Kim et al., 2019; Kitzerow et al., 2016]. These studies, however, have not been large enough to be clearly decisive as to the best metric, and these differential results may be due to different participant populations, interventions or lengths of treatments. At 13 months, our intervention period was longer than that of Kim et al. [2019], but similar to Kitzerow et al. [2016], in which there was a trend for significance, a medium ES and the z‐standardized change scores of the ADOS CSS and ADOS‐BOSCC did not differ. This suggests that in longer intervention trials (~12 months), the ADOS and ADOS‐BOSCC can both be sensitive to change over time.

#### *Intervention effects*

Contrary to expectation, the ADOS‐BOSCC did not produce a larger ES than either of the ADOS metrics. Differences between the three total score ES were not significant. On the ADOS, those with “few to no words” at baseline demonstrated significant benefit from the PACT intervention in contrast to minimal improvement within the TAU group, resulting in a moderate significant ES (Table 3). In contrast, those with “some words” at baseline improved to a similar extent regardless of receiving PACT or TAU. The ES for the two sub‐groups were not significantly different but this pattern may be important to consider further in future trials of PACT. Possible explanations include that earlier PACT therapy stages aimed at children with no words, may be more distinguishable from and beneficial than TAU compared to later stages, aimed at children who are already developing language [see PACT therapy manual in supplementary materials of Green et al., 2010]. Alternatively, the children with some words at baseline may have been likely to improve regardless of what therapy they received. However, the fact that the Module 2 children, with their relatively greater language ability, demonstrated advantage from receiving PACT over TAU may be inconsistent with this interpretation. The exploratory nature and lack of significant difference between the two groups limits the conclusions that can be drawn here. Further exploration of this in future PACT trials will be of interest, in line with calls for our field to better understand who benefits most from different therapies [e.g. Simonoff, 2018].

Though not significantly different, the ES for CSS was about half the size of the ADOS algorithm effect, potentially suggesting some degree of sensitivity is lost in the transition from algorithm to CSS. This is likely related in part to the lower pre‐post correlation for the ADOS CSS, resulting from the compacted baseline score range, which reduces the power of the analysis. Researchers should consider this alongside other relative merits and challenges of the two ADOS metrics.

Regarding the lack of a larger ES for ADOS‐BOSCC, it may be that the shorter capture of behavior (12 min) compared to the full ADOS assessment limits the change that is evidenced. It may also be that some behaviors are not captured when scoring from 10‐year‐old videos. It may be that the standard naturalistic BOSCC would capture a greater degree of change as the structured nature of the ADOS tasks may be influencing the range of behaviors and degree of change detected. Though Kim et al. [2019] report a strong correlation in the overall change scores of the two BOSCC versions, change detected in RRB varied across the two methods. Further research using the ADOS‐BOSCC and standard BOSCC are needed in order to explore any relative differences.

### *Full* *PACT* *Sample* *ADOS*

For the full PACT sample, inclusive of Module 1 and Module 2 children, the ADOS algorithm detected an overall significant and moderate ES and the ADOS CSS had an overall significant small ES (Table S13). The results of the subdomains suggested improvements in RRB were an important part of this effect, especially among Module 2 children. The Module 2 item‐level results provide further evidence for this.

### *PACT* *“Signature of Change”*

#### *Module 1* *ADOS‐BOSCC* *and* *ADOS*

Presented for illustration, but suggested for future larger trials and systematic reviews, the item‐level analyzes gave some weak non‐significant evidences that Module 1 children who received PACT improved in their use of nonverbal communication behaviors (Fig. 3). Use of Gesture was one of the largest improving items on both the ADOS‐BOSCC and ADOS, while Use of Facial Expressions was a notable improvement on the ADOS. Intonation was the largest improver on the ADOS but with large confidence intervals due to the smaller subsample in use for this item. These changes are in line with the goals and strategies used in PACT to improve children's communicative initiations. Given that these children start at limited levels of communication, it makes sense for nonverbal communication behaviors to be among the first behaviors to improve. Nonverbal communication behaviors are predictive of later language and social interaction [Stone, Ousley, Yoder, Hogan, & Hepburn, 1997].

#### *Module 2* *ADOS*

Module 2 children showed large and significant improvements on Mannerisms and Repetitive Interests/Stereotyped Behaviors on the ADOS, and a medium but not significant improvement on rapport (Fig. S4). The item plot therefore provided more specific evidence that the PACT intervention “signature of change” is associated with effects that are equally as strong for behaviors within the RRB subdomain as for certain social communication skills. The finding is surprising in that across ADOS and ADOS‐BOSCC, we had lower inter‐rater reliability in the RRB subdomain. This lower reliability may be related to the behaviors being infrequent or harder to reliably identify, as previously suggested by the BOSCC developers [Grzadzinski & Lord, 2018], and should therefore be interpreted with caution.

One potential explanation is that the improvement in rapport may mean that the interaction between researcher and child is more comfortable and less anxiety provoking, perhaps reducing the use of RRBs for self‐regulation [Rodgers, Riby, Janes, Connolly, & McConachie, 2012]. This may be particularly the case for Module 2 children on account of their higher language levels. No such hypotheses have yet been directly tested. The intervention “signature of change” item‐level plots have advanced our understanding of the impact of PACT, enhancing understanding of the therapeutic mechanism and the need to better target some specific skills and behaviors. Such analyzes, particularly using data pooled across trials, would be useful for therapeutic development.

### *Research Implications*

We focused here on sensitivity to change of the ADOS metrics and ADOS‐BOSCC and the profile of change across individual behaviors following the PACT therapy. The “signature of change” plots may be valuable for intervention development and as part of systematic reviews, but do not replace the need for clear prespecified primary outcomes. Contrary to concerns that the ADOS may not be an appropriate outcome measure on account of being designed for diagnosis [Anagnostou et al., 2015], the ADOS algorithm evidenced a significant intervention effect.

The ADOS and ADOS‐BOSCC measure a range of social communication skills and repetitive behaviors and restricted interests during structured interaction with a researcher. Within parent‐mediated social communication interventions, such blind‐rated observational assessments with a non‐trained interaction partner are important outcome measures to assess whether target skills have generalized beyond the intervention context [Carruthers, Pickles, Slonims, Howlin, & Charman, 2020; Sandbank et al., 2020]. However, there is a need to consider how best to pair the methodological strengths of measures such as the ADOS and BOSCC, with the priorities of the autistic community and parents [Lai, Anagnostou, Wiznitzer, Allison, & Baron‐Cohen, 2020]. One suggestion has been to assess social interaction with siblings, for instance with the naturalistic BOSCC, which permits exploration of relationship with family members, a priority for parents, and does not risk correlated measurement error [McConachie et al., 2018; Sandbank et al., 2020]. Likewise, it is important to consider the targets of interventions in light of the views of autistic individuals and their parents [Fletcher‐Watson, 2018; Kapp et al., 2019]. The relative advantages between the naturalistic BOSCC and the structured ADOS‐BOSCC and ADOS are yet to be fully understood.

To construct a measure for providing evidence of response to intervention requires more than just consideration of the internal psychometrics. High test–retest reliability and larger item scoring ranges can characterize both well measured traits likely unresponsive to intervention and well‐constructed measures of behavior thought to be responsive. What is important for measures that span heterogeneous domains such as autism is a relatively greater focus on the “lead” behaviors likely to respond first to the kinds of therapeutic interventions being considered. As others have highlighted, there is unlikely to be a “one size fits all” solution to finding an optimal outcome measure across all autism interventions [Grzadzinski et al., 2020]. Grzadzinski et al. [2020] recommend researchers consider which behaviors will likely change as a result of a particular intervention and how broad that impact is likely to be (e.g., across many social communication behaviors or in specific behaviors). To do this with confidence requires a comprehensive understanding of the development of social‐communication of autistic children and of what impact different therapeutic approaches have. Use of tools such as the BOSCC and plots of item‐level effects such as the ones we have presented can provide critical insight to advance this understanding.

### *Limitations*

Although the ADOS assessments were coded blind to treatment group, they were not coded blind to timepoint, which may introduce some bias. The PACT trial, designed before development of the CSS, administered the same ADOS Module at baseline and endpoint. As a consequence some children received an endpoint ADOS administration that was not optimally aligned with their language level. Although some caution is thus needed with the interpretation of our endpoint scores across the three metrics, this is unlikely to explain the pattern of our results. In addition, it should be noted that the ES reported above are in line with common practice where variance of the pooled sample at baseline is the denominator. Those reported in Table S12, where variance in the change is used, suggest a more modest ES for the ADOS. This has a greater influence on the ADOS as the sample had a small variance at baseline as a result of the eligibility criteria (i.e., all children had to receive a diagnosis of core autism on the ADOS). Inter‐rater reliability, item‐rest correlations, and factor loadings were lower among some items in the ADOS‐BOSCC, particularly for the RRB subdomain, which also had lower inter‐rater reliability on the ADOS. All analyzes reported are post‐hoc to the original trial and multiple testing considerations would suggest that these analyzes are underpowered for robust interpretation. Despite this, these analyzes have been informative. Exploratory secondary analyzes are important to conduct and discuss if we are to maximize the knowledge that can be gained from trials, though pre‐registration, careful reporting, and caution with over‐interpretation are important [Furberg & Friedman, 2012].

## Conclusions

The ADOS‐BOSCC had strong psychometric properties but did not evidence a larger intervention effect than the ADOS. Our study has suggested that the ADOS can be sensitive to change and able to evidence a significant intervention effect when used in RCTs for longer intervention durations, in this case particularly for Module 2 children. Exploration of the item‐level intervention “signature of change” suggests it as a potentially informative analysis to further our understanding of what specific behaviors are impacted by interventions, and in considering potential mechanisms. Other intervention trials may benefit from doing the same.

## Supporting information


**Appendix** S1. Additional Details for Methods.
**Figure S1.** Box plots with scatter of ADOS‐BOSCC social communication, ADOS algorithm social affect, and ADOS CSS social affect at baseline and endpoint across intervention groups for Module 1.
**Figure S2.** Box plots with scatter of ADOS‐BOSCC RRB, ADOS algorithm RRB, and ADOS CSS RRB at baseline and endpoint across intervention groups for Module 1.
**Figure S3.** Forest plot of intervention effect size estimates for the ADOS algorithm and ADOS CSS total and subdomain scores for the full PACT sample.
**Figure S4.** Forest plot of intervention ‘signature of change’: effect estimates for the items of the ADOS (Module 2) with 95% confidence intervals corrected for multiple comparisons within each measure with effect sizes.
**Table S1.** Baseline Characteristics of Module 2 Children by Intervention Group
**Table S2.** Mean Values of Parent‐Reported Vineland Language Measures at Baseline and Endpoint by Group for Module 1 Children
**Table S3.** Intra‐Class Correlations and Item‐Rest Correlations for ADOS‐BOSCC Module 1 Items
**Table S4.** Confirmatory Factor Loadings on the ADOS‐BOSCC Module 1 for the Two‐Factor Solution
**Table S5.** Baseline and Change Score Correlations between ADOS‐BOSCC, ADOS algorithm, ADOS CSS and Measures of Cognitive and Language Skills for Module 1
**Table S6.** Pearson Correlations between Social Communication Subscales and RRB Subscales of the ADOS‐BOSCC, ADOS Algorithm Scores, and ADOS CSS for Module 1 Children
**Table S7.** Pre‐post Change Scores for ADOS Module 1 Total and Subdomain Scores for Children with Few to No Words and Some Words at Baseline by Intervention Group
**Table S8.** Comparison of Effect Sizes from Module 1 Intervention Effect Analysis with Bootstrapping
**Table S9.** Intervention Effect Results for ADOS‐BOSCC, ADOS Algorithm, and ADOS CSS for Module 1 using ANCOVA (Regress)
**Table S10.** Intervention Effect Results for ADOS‐BOSCC Social Communication, ADOS Algorithm Social Affect, and ADOS CSS Social Affect for Module 1
**Table S11.** Intervention Effect Results for ADOS‐BOSCC RRB, ADOS Algorithm RRB, and ADOS CSS RRB for Module 1
**Table S12.** Effect Sizes [95% CI] for Intervention Models using Change Score Variation
**Table S13**. Pre‐post Change Scores (SD) for ADOS‐BOSCC and ADOS by Intervention Group for Full PACT Sample
**Table S14.** Intervention Effect Results for ADOS Algorithm and ADOS CSS for the Full PACT Sample
**Table S15.** Intervention Effect Results for ADOS Algorithm and ADOS CSS Social Affect Subdomains for the Full PACT Sample
**Table S16.** Intervention Effect Results for ADOS Algorithm and ADOS CSS RRB Subdomains for the Full PACT Sample
