# Supplementary material for: Utility of the Autism Diagnostic Observation Schedule and the Brief Observation of Social and Communication Change for Measuring Outcomes for a Parent‐Mediated Early Autism Intervention
Source: Autism Res. 2020 Dec 4;14(2):411–25. doi: 10.1002/aur.2449 (PMC7898818; doi:10.1002/aur.2449)
Supplement: Supplementary file 1 — Appendix S1. Additional Details for Methods. Figure S1. Box plots with scatter of ADOS‐BOSCC social communication, ADOS algorithm social affect, and ADOS CSS social affect at baseline and endpoint across intervention groups for Module 1. Figure S2. Box plots with scatter of ADOS‐BOSCC RRB, ADOS algorithm RRB, and ADOS CSS RRB at baseline and endpoint across intervention groups for Module 1. Figure S3. Forest plot of intervention effect size estimates for the ADOS algorithm and ADOS CSS total and subdomain scores for the full PACT sample. Figure S4. Forest plot of intervention ‘signature of change’: effect estimates for the items of the ADOS (Module 2) with 95% confidence intervals corrected for multiple comparisons within each measure with effect sizes. Table S1. Baseline Characteristics of Module 2 Children by Intervention Group Table S2. Mean Values of Parent‐Reported Vineland Language Measures at Baseline and Endpoint by Group for Module 1 Children Table S3. Intra‐Class Correlations and Item‐Rest Correlations for ADOS‐BOSCC Module 1 Items Table S4. Confirmatory Factor Loadings on the ADOS‐BOSCC Module 1 for the Two‐Factor Solution Table S5. Baseline and Change Score Correlations between ADOS‐BOSCC, ADOS algorithm, ADOS CSS and Measures of Cognitive and Language Skills for Module 1 Table S6. Pearson Correlations between Social Communication Subscales and RRB Subscales of the ADOS‐BOSCC, ADOS Algorithm Scores, and ADOS CSS for Module 1 Children Table S7. Pre‐post Change Scores for ADOS Module 1 Total and Subdomain Scores for Children with Few to No Words and Some Words at Baseline by Intervention Group Table S8. Comparison of Effect Sizes from Module 1 Intervention Effect Analysis with Bootstrapping Table S9. Intervention Effect Results for ADOS‐BOSCC, ADOS Algorithm, and ADOS CSS for Module 1 using ANCOVA (Regress) Table S10. Intervention Effect Results for ADOS‐BOSCC Social Communication, ADOS Algorithm Social Affect, and ADOS CSS Social Affect for Module 1 Table [file AUR-14-411-s001.docx]

**Utility of the Autism Diagnostic Observation Schedule and the Brief Observation of Social and Communication Change as Measures of Outcomes for a Parent-Mediated Early Autism Intervention**

**Supplementary material**

**Methods**

For full details of the PACT study, see Green et al. (2010).

**Sample (further details)**

Diagnosis was confirmed according to the international standard tests (social and communication domains of the ADOS-G, and two of three domains of the Autism Diagnostic Interview Revised [ADI-R] algorithm). Children were excluded if they had a twin with autism; had a non-verbal age equivalent to 12 months or younger on the Mullen Early Learning Scales; epilepsy requiring medication; severe hearing or visual impairment in a parent or the child; or a parent with a severe psychiatric disorder requiring treatment. At home, participating parents spoke English with their child.

**ADOS (further details)**

Most items are initially scored between 0 (autistic characteristic is not present) to 2 or 3 (autistic characteristic is present) with scores of 3 becoming 2 for the algorithm scoring. The coders participated in regular coding meetings to ensure maintenance of research-level reliability. At baseline, 74 (63%) module 1 children had ‘Few to no words’ and were scored on ‘Response to Joint Attention’ and ‘Intonation of Vocalizations or Verbalizations’ but not ‘Pointing’ or ‘Stereotyped/Idiosyncratic Use of Words or Phrases’, and vice versa for the remaining 43 (37%) module 1 children who had ‘Some words’. At endpoint 58 (52%) module 1 children had ‘Few to no words’ and 53 (48%) had ‘Some words’ and were scored with corresponding items.

**ADOS-BOSCC (further details)**

The ADOS tasks coded with the ADOS-BOSCC are those that most closely resemble the more naturalistic free play and bubble play administration of the standard BOSCC. The Response to Name activity is excluded from the segment if it occurs during Free Play or Bubble Play. At least some time from RJA was added to Segment A in 92.3% of the coded observations. At least some time from Snack was added to Segment B to 89.9% of the coded observations. The selected clips were coded from the beginning of the task, with the exception of Free Play, which was coded after the child was able to explore the toys for one minute.

SC was trained to reliability by the developers of the BOSCC and then in turn trained YK, NH and RR, three MSc students. In line with suggestions of the developers, we were successful in training to reliability post-graduate students with no previous autism knowledge, but training was time consuming and took 15-25 videos which is up to double the number given as a guide (Grzadzinski & Lord, 2018). Fewer tapes may be needed for individuals already familiar with identifying autism characteristics. Coders were deemed reliable after coding three consecutive videos (six segments) in which the total score of all 16 items was within five points of the total score reached by consensus, and where at least 13 of the 16 items were within one point of the consensus item scores. Coded over a period of six months during which fortnightly consensus meetings were held to maintain reliability, videos were randomly allocated to the four coders stratified for timepoint and group. The same participant was not coded by the same coder at baseline and endpoint. Codes from videos used during consensus meetings were entered from the coder the video had originally been allocated.

**Other Measures**

***Mullen Scales of Early Learning* (MSEL)**

The visual reception and fine motor scales of the examiner administered MSEL *(Mullen, 1995)* were used to quantify non-verbal developmental abilities in the children at baseline only, with a non-verbal age equivalent score computed as the average of these domains. Construct, convergent, and divergent validity has been established with autism samples (Swineford, Guthrie, & Thurm, 2015).

***Pre-school language scales***

The PLS (Zimmerman, Steiner, Pond, Boucher, & Lewis, 1997) was used to obtain standard expressive language scores. The value from baseline is used as a covariate in the intervention models, as per the original analysis (2010).

***Vineland Adaptive Behaviour Scales.***

The parent survey version, *second edition (Sparrow, Cicchetti, & Balla, 2005),* was used at baseline and endpoint to measure adaptive functioning. Subdomain age equivalent scores for expressive and receptive language are used for our analyses. Validity has been established with autism samples (de Bildt, Kraijer, Sytema, & Minderaa, 2005; Perry & Factor, 1989) and it is considered to have adequate reliability, validity and responsiveness to quantify social communication skills in clinical trials of interventions for autism (Anagnostou et al., 2015).

***Family and intervention-related factors***

Demographic data were collected via semi-structured parent interview and questionnaire responses, and the following were dichotomous variables were coded for analysis: family composition (dual vs. single parent status), ethnicity (both parents white vs. one/both parents non-white), household income (less than vs. equal to or greater than £40,000 per annum) and parental educational attainment (less than vs. equal to or greater than one parent with some qualification post 16 years of age). Among families assigned to the PACT arm, the number of intervention sessions attended by families was also recorded for a measure of PACT dose.

**Data analysis**

Of the 117 Module 1 ADOS assessments, six (4%) of the participants did not complete an ADOS assessment at endpoint, resulting in 111 complete pairs of baseline and endpoint ADOS assessments; 57 in PACT and 54 in TAU. A further seven ADOS assessments were not able to be coded with the ADOS-BOSCC as a result of technical issues with the video files or insufficient material that could be coded.

Of the 152 participants within the full PACT sample, six of the participants did not complete an ADOS assessment at endpoint. When estimated by full information maximum likelihood the SEM intervention model of the main paper allows for the inclusion of observations missing baseline or endpoint on the assumption of a missing-at-random data mechanism.

**Changes to pre-registered analysis**

The pre-registered analysis was revised primarily in relation to the exclusion of the ADOS-BOSCC data for the Module 2 children, as the coding scheme used for Module 2 was a development version that was not subsequently adopted by the ADOS-BOSCC developers. Our analysis of the ADOS-BOSCC was therefore restricted to Module 1 children, this analysis being in all other respects as prespecified. For the examination of Module 1 and Module 2 children for the ADOS, subsequent to the initial analysis, two revisions were made. First the addition of an analysis of the trial data as scored in the ADOS-CSS. Second, in order to match the strata used in the mapping of the ADOS algorithm scores to the ADOS CSS, the analyses of the ADOS algorithm scores was stratified into Module 1 No-words, Module 1 Some Words and Module 2 children, with a combined effect size for these strata being obtained by a variance minimizing combination.

**Results**

**Table S1**

*Baseline Characteristics of Module 2 Children by Intervention Group*

|  | PACT (n = 17) | TAU (n = 18) |
| --- | --- | --- |
| Child age (months; mean, range) | 51 (41-59) | 50 (31-58) |
| Girl | 2 (12%) | 2 (11%) |
| Parents’ ethnic origin  Both white  Mixed*  Non-white | 12 (71%)  3 (18%)  2 (12%) | 13 (72%)  1 (6%)  4 (22%) |
| Education (one parent with qualifications after age 16y) | 14 (83%) | 11 (61%) |
| Socioeconomic status † | 12 (71%) | 10 (56%) |
| Mullen non-verbal age equivalent (months; mean, SD) | 41.5 (8.31) | 37.1 (9.86) |

*Note.* Data are number (%), unless otherwise indicated. PACT = Preschool Autism Communication Trial. TAU = treatment as usual.

*One white parent and the other non-white. † Dichotomised as at least one parent in professional or administrative occupation versus all others.

**Table S2**

*Mean Values of Parent-Reported Vineland Language Measures at Baseline and Endpoint by Group for Module 1 Children*

| Measure | Baseline |  | Outcome |  |
| --- | --- | --- | --- | --- |
|  | PACT  M (SD) | TAU  M (SD) | PACT  M (SD) | TAU  M (SD) |
| Expressive age equivalent (months) | 14.5 (6.74)  n = 57 | 14.4 (8.64)  n = 55 | 20.8 (11.9)  n = 53 | 18.9 (9.46)  n = 51 |
| Receptive age equivalent (months) | 12.7 (6.99)  n = 60 | 12.3 (7.24)  n = 55 | 24.5 (18.6)  n = 53 | 19.1 (10.3)  n = 51 |

*Note.* Scores are from the Vineland Adaptive Behaviour Scales – second edition, parent survey. PACT = Preschool Autism Communication Trial; TAU = treatment as usual

**Table S3**

*Intra-Class Correlations and Item-Rest Correlations for ADOS-BOSCC Module 1 Items*

| Item | IRR across 4 coders [95% CI] | Item-Rest Correlation within SC subscale | Item-Rest Correlation within RRB subscale |
| --- | --- | --- | --- |
| 1 – eye contact | .48 [.20, .77] | 0.43 |  |
| 2 – facial expressions | .89 [.77, .96] | 0.33 |  |
| 3 – gestures | .79 [.59, .92] | 0.47 |  |
| 4 – vocalisations | .93 [.84, .97] | 0.56 |  |
| 5 – integration | .88 [.74, .96] | 0.78 |  |
| 6 – social overtures | .64 [.37, .86] | 0.65 |  |
| 7 – social responses | .50 [.23, .78] | 0.63 |  |
| 8 – social engagement | .73 [.46, .90] | 0.70 |  |
| 9 – requesting | .69 [.45, .88] | 0.78 |  |
| 10 – play with objects | .64 [.38, .86] |  | 0.30 |
| 11 – sensory | .76 [.54, .91] |  | 0.41 |
| 12 – mannerisms | .46 [.17, .76] |  | 0.14 |
| 13 - RRB | .51 [.24, .79] |  | 0.29 |
| 14 – activity level | .54 [.26, .81] |  |  |
| 15 – irritability/disruption | .70 [.46, .89] |  |  |
| 16 - anxiety | .64 [.38, .86] |  |  |

*Note.* Item-rest correlation is the correlation between each item and the subscale score excluding that item. All item-rest correlations were calculated using baseline data.

RRB = Restricted and Repetitive Behaviour subscale; SC = Social Communication subscale

**Table S4**

*Confirmatory Factor Loadings on the ADOS-BOSCC Module 1 for the Two-Factor Solution*

| Factor 1 –  Social communication | Loading | Factor 2 – Restricted/repetitive behaviour | Loading |
| --- | --- | --- | --- |
| Eye contact - A | .41 | Play – A | .60 |
| Eye contact - B | .60 | Play – B | .80 |
| Facial expressions - A | .43 | Sensory interests - A | .38 |
| Facial expressions - B | .46 | Sensory interests - B | .25 |
| Gestures - A | .63 | Mannerisms - A | .07 |
| Gestures - B | .56 | Mannerisms - B | .09 |
| Vocalizations - A | .81 | Repetitive/Stereotyped - A | .42 |
| Vocalizations - B | .79 | Repetitive/Stereotyped - B | .33 |
| Integration - A | .88 |  |  |
| Integration - B | .79 |  |  |
| Social overtures - A | .72 |  |  |
| Social overtures - B | .73 |  |  |
| Social responses - A | .57 |  |  |
| Social responses - B | .75 |  |  |
| Engagement - A | .69 |  |  |
| Engagement - B | .84 |  |  |
| Requesting – A | .79 |  |  |
| Requesting – B | .66 |  |  |

*Note.* Of the 234 possible observations (baseline and endpoint data), there were 12 observations missing. One further observation only had data for one segment. Final sample is 222 observations from 117 participants. A – Segment A; B – Segment B

**Table S5**

*Baseline and Change Score Correlations between ADOS-BOSCC, ADOS algorithm, ADOS CSS and Measures of Cognitive and Language Skills for Module 1*

|  | Correlations between baseline values | | | Correlations between change scores | | |
| --- | --- | --- | --- | --- | --- | --- |
| Measure | ADOS-BOSCC | ADOS algorithm | ADOS CSS | ADOS-BOSCC | ADOS algorithm | ADOS CSS |
| ADOS-BOSCC | - |  |  | - |  |  |
| ADOS algorithm | 0.68** | - |  | 0.51** | - |  |
| ADOS CSS | 0.43** | 0.82** | - | 0.41** | 0.88** | - |
| Nonverbal IQ | -0.55** | -0.50** | -0.27** | - | - | - |
| Expressive language | -0.50** | -0.43** | -0.19 | 0.35** | 0.28** | 0.16 |
| Receptive language | -0.33** | -0.21* | -0.008 | 0.41** | 0.19 | 0.10 |

*Note.* Spearman correlations were conducted with nonverbal IQ, expressive language and receptive language variables. *n=*104 except expressive language [baseline *n=*99, change score *n*=93] and receptive language [baseline *n=*102, change score *n=*96]. ADOS = Autism Diagnostic Observation Schedule; ADOS-BOSCC = Brief Observation for Social Communication Change–version for ADOS; CSS = Calibrated Severity Scores

** p<.01 * p<.05

**Table S6**

| Measure/Subdomain |  | Social Communication/Affect | | |  | RRB | | |
| --- | --- | --- | --- | --- | --- | --- | --- | --- |
|  |  | ADOS-BOSCC | ADOS algorithm | ADOS  CSS |  | ADOS-BOSCC | ADOS algorithm | ADOS CSS |
| **BASELINE SCORES** | **Social Communication/Affect** |  |  |  | **RRB** |  |  |  |
| ADOS-BOSCC |  | - |  |  |  | - |  |  |
| ADOS algorithm |  | 0.61**  (*n* = 104) | *-* |  |  | 0.46**  (*n* = 104) | - |  |
| ADOS CSS |  | 0.31**  (*n* = 104) | 0.78**  (*n* = 104) | - |  | 0.41**  (*n* = 104) | 0.93**  (*n* = 104) | - |
| **CHANGE SCORES** |  |  |  |  |  |  |  |  |
| ADOS-BOSCC |  | - |  |  |  | - |  |  |
| ADOS algorithm |  | 0.49**  (*n* = 104) | *-* |  |  | 0.20*  (*n* = 104) | - |  |
| ADOS CSS |  | 0.34**  (*n* = 104) | 0.87**  (*n* = 104) | - |  | 0.16  (*n* =104) | 0.94**  (*n* = 104) | - |

*Pearson Correlations between Social Communication Subscales and RRB Subscales of the ADOS-BOSCC, ADOS Algorithm Scores and ADOS CSS for Module 1 Children*

*Note.* ADOS = Autism Diagnostic Observation Schedule, ADOS-BOSCC = Brief Observation for Social Communication Change – version for ADOS, SA = social affect, SC = social communication, RRB = restricted and repetitive behaviours. ** p <.01 * p <.05

**Figure S1.** Box Plots with Scatter of ADOS-BOSCC Social Communication, ADOS Algorithm Social Affect and ADOS CSS Social Affect at Baseline and Endpoint across Intervention Groups For Module 1. ADOS = Autism Diagnostic Observation Schedule; ADOS-BOSCC = Brief Observation of Social and Communication Change-version for ADOS; PACT = Preschool Autism Communication Trial; TAU = Treatment As Usual

**Figure S2.** Box Plots with Scatter of ADOS-BOSCC RRB, ADOS algorithm RRB and ADOS CSS RRB at Baseline and Endpoint across Intervention Groups for Module 1. ADOS = Autism Diagnostic Observation Schedule; ADOS-BOSCC = Brief Observation of Social and Communication Change-version for ADOS; PACT = Preschool Autism Communication Trial; TAU = Treatment As Usual

**Table S7**

*Pre-post Change Scores for ADOS Module 1 Total and Subdomain Scores for Children with Few to No words and Some words at Baseline by Intervention Group*

|  | Baseline | | Endpoint | | TAU change | | PACT change | |
| --- | --- | --- | --- | --- | --- | --- | --- | --- |
|  | Mean (SD) | | Mean (SD) | | Mean difference (SD) | Effect size (*d_z_*) | Mean difference (SD) | Effect size (*d_z_*) |
|  | TAU | PACT | TAU | PACT |  |  |  |  |
| Few to no words total  Some words total | 21.9 (2.72)  19.4 (2.78) | 23.0 (3.21)  17.4 (2.45) | 21.4 (3.79)  16.4 (5.21) | 19.1 (5.70)  14.9 (4.42) | -0.44 (3.65)  -3.00 (4.77) | 0.12  0.63 | -3.88 (5.08)  -2.56 (4.37) | 0.76  0.58 |
| Few to no words SA  Some words SA | 17.0 (2.06)  14.8 (2.28) | 17.2 (2.31)  13.6 (2.70) | 16.4 (2.97)  11.9 (4.50) | 14.4 (4.80)  10.9 (3.66) | -0.56 (2.92)  -2.95 (3.98) | 0.19  0.74 | -2.79 (4.58)  -2.72 (4.47) | 0.61  0.61 |
| Few to no words RRB  Some words RRB | 4.91 (1.84)  4.55 (1.28) | 5.79 (1.79)  3.83 (1.65) | 5.03 (1.82)  4.50 (1.91) | 4.71 (1.66)  4.00 (1.97) | 0.13 (2.28)  -0.05 (2.04) | -0.05  0.02 | -1.09 (1.94)  0.17 (1.76) | 0.56  -0.09 |

*Note.* A negative change score indicates improvement on the scale. Paired sample t-tests tested within-subdomain change. ADOS = Autism Diagnostic Observation Schedule, *d_z_ =* Cohen’s d_z_ effect size for correlated samples, PACT = Preschool Autism Communication Trial, SA = social affect, RRB = restricted and repetitive behaviours, TAU = treatment-as-usual. *n*=104 (TAU n = 52 [no words *n* = 32, some words *n* = 20]; PACT n = 52 [no words *n* = 34, some words *n* = 18).

*p<.05, ** p<.01

**Table S8.**

*Comparison of Effect Sizes from Module 1 Intervention Effect Analysis with Bootstrapping*

| Effect size comparison | Estimate of difference | Bootstrap standard error | *p* | Confidence Intervals |
| --- | --- | --- | --- | --- |
| ADOS vs ADOS-BOSCC | 0.20 | 0.35 | 0.561 | -0.48, 0.88 |
| CSS vs ADOS-BOSCC | 0.02 | 0.18 | 0.895 | -0.32, 0.37 |
| CSS vs ADOS | -0.18 | 0.28 | 0.526 | -0.73, 0.37 |

*Note.* ADOS = Autism Diagnostic Observation Schedule; ADOS-BOSCC = Brief Observation of Social Communication Change-version for ADOS; CSS = Calibrated Severity Scores

**Table S9**

*Intervention Effect Results for ADOS-BOSCC, ADOS Algorithm and ADOS CSS for Module 1 using ANCOVA (regress)*

|  | ADOS-BOSCC | | ADOS algorithm | | | | ADOS CSS | | |  |
| --- | --- | --- | --- | --- | --- | --- | --- | --- | --- | --- |
| Strata |  |  | Few to no words | | Some words | | |  |  | |
|  | Coefficient | 95% CI | Coefficient | 95% CI | Coefficient | 95% CI | | Coefficient | 95% CI | |
| PACT intervention | -2.13 | -5.27, 1.01 | -2.18 | -4.55, 0.19 | 0.25 | -3.05, 3.56 | | -0.37 | -0.98, 0.25 | |
| Covariates |  |  |  |  |  |  | |  |  | |
| Nonverbal IQ | -0.43* | -0.81, -0.05 | -0.28 | -0.60, 0.05 | -0.26 | -0.56, 0.03 | | -0.11* | -0.18, -0.04 | |
| Expressive language | -0.59* | -1.06, -0.12 | -0.21 | -0.66, 0.25 | 0.33 | -0.21, 0.88 | | 0.01 | -0.07, 0.10 | |
| London vs Manchester | -2.14 | -5.75, 1.48 | -3.90* | -6.32, -1.47 | -0.73 | -5.09, 3.62 | | -0.91* | -1.61, -0.20 | |
| London vs Newcastle | -2.42 | -6.50, 1.66 | -1.58 | -4.47, 1.32 | -1.14 | -5.23, 2.94 | | -0.74 | -1.53, 0.04 | |
| Sex | 2.18 | -2.93, 7.28 | 1.13 | -2.45, 4.71 | -1.24 | -7.04, 4.56 | | 0.40 | -0.60, 1.40 | |
| Age group | 0.34 | -2.95, 3.62 | 1.17 | -0.98, 3.31 | -1.36 | -4.97, 2.25 | | -0.15 | -0.78, 0.48 | |
| Parent’s job | 1.58 | -1.86, 5.03 | 1.03 | -1.23, 3.28 | -1.21 | -5.66, 3.24 | | 0.29 | -0.38, 0.96 | |
| Parent’s education | 1.08 | -2.77, 4.93 | 0.72 | -1.94, 3.37 | 4.41 | -0.59, 9.41 | | 0.19 | -0.56, 0.95 | |
| Effect size (95% CI) | -0.24 (-0.59, 0.11) | | -0.46* (-0.88, -0.04) | | 0.06 (-0.59, 0.71) | | | -0.26 (-0.70, 0.17) | | |
|  |  | | -0.31 (-0.66, 0.04) | | | | |  | | |

*Note.* Negative effect sizes are in favour of PACT. Effect sizes are calculated using the pooled standard deviation at baseline. ADOS = Autism Diagnostic Observation Schedule; ADOS-BOSCC = Brief Observation of Social Communication Change-version for ADOS; CSS = Calibrated Severity Scores

* p<.05 ** p<.001

**Table S10**

*Intervention Effect Results for ADOS-BOSCC Social Communication, ADOS Algorithm Social Affect and ADOS CSS Social Affect for Module 1*

|  | ADOS-BOSCC  Social Communication | | ADOS Algorithm  Social Affect | | | | ADOS CSS  Social Affect | |
| --- | --- | --- | --- | --- | --- | --- | --- | --- |
| Strata at baseline |  |  | Few to no words | | Some words | |  |  |
|  | Coefficient | 95% CI | Coefficient | 95% CI | Coefficient | 95% CI | Coefficient | 95% CI |
| PACT intervention | -1.85 | -4.30, 0.61 | -1.31 | -3.11, 0.51 | -.002 | -2.36, 2.35 | -0.41 | -1.07, 0.26 |
| Covariates |  |  |  |  |  |  |  |  |
| Cognitive ability (nonverbal) | -0.49* | -0.79, 0.20 | -0.12 | -0.38, 0.15 | -0.26* | -0.47, -0.06 | -0.10* | -0.18, -0.03 |
| Expressive language | -0.75** | -1.11, -0.39 | -0.35 | -0.71, 0.002 | 0.20 | -0.21, 0.61 | 0.05 | -0.04, 0.15 |
| London vs Manchester | -1.52 | -4.52, 1.48 | -3.18* | -5.15, -1.21 | -1.11 | -4.38, 2.16 | -1.13 | -1.92, -0.34 |
| London vs Newcastle | -0.72 | -4.06, 2.62 | -1.83 | -4.19, 0.53 | -0.68 | -3.72, 2.35 | -0.84 | -1.72, 0.04 |
| Sex | -0.10 | -4.35, 4.14 | -0.14 | -3.07, 2.78 | -0.09 | -4.16, 3.98 | -0.13 | -1.25, 0.99 |
| Age group | 0.74 | -1.93, 3.40 | 0.13 | -1.60, 1.87 | -1.36 | -4.03, 1.31 | -0.52 | -1.23, 0.18 |
| Parent’s job | 1.93 | -0.93, 4.80 | 0.98 | -0.86, 2.82 | -1.13 | -4.43, 2.18 | 0.20 | -0.56, 0.96 |
| Parent’s education | 0.69 | -2.49, 3.88 | -0.38 | -2.53, 1.77 | 2.95 | -0.84, 6.73 | -0.05 | -0.89, 0.80 |
| Effect size (95% CI) |  | | -0.60 (-1.43, 0.23) | | -0.0008 (-0.93, 0.93) | |  | |
|  | -0.27 (-0.62, 0.09) | | -0.33 (-0.96, 0.29) | | | | -0.29 (-0.76, 0.18) | |

*Note.* Negative effect sizes are in favour of PACT. Cognitive ability (nonverbal) is measured with the Mullen Scales for Early Learning. Expressive language is measured with the Preschool Language Scales. Effect sizes are calculated using the pooled standard deviation at baseline. ADOS = Autism Diagnostic Observation Schedule; ADOS-BOSCC = Brief Observation of Social Communication Change-version for ADOS. *n* = 104, * p < .05 ** p < .001

**Table S11**

*Intervention Effect Results for ADOS-BOSCC RRB, ADOS Algorithm RRB and ADOS CSS RRB for Module 1*

|  | ADOS-BOSCC  RRB | | ADOS Algorithm  RRB | | | | ADOS CSS  RRB | | |  |
| --- | --- | --- | --- | --- | --- | --- | --- | --- | --- | --- |
|  |  |  | Few to no words | | Some words | | |  |  | |
|  | Coefficient | 95% CI | Coefficient | 95% CI | Coefficient | 95% CI | | Coefficient | 95% CI | |
| PACT intervention | -0.34 | -1.48, 0.79 | -0.44 | -1.19, 0.31 | -0.02 | -1.12, 1.08 | | -0.37 | -0.99, 0.25 | |
| Covariates |  |  |  |  |  |  | |  |  | |
| Cognitive ability (nonverbal) | -0.22* | -0.35, -0.09 | -0.21** | -0.31, -0.11 | -0.12* | -0.22, -0.02 | | -0.13** | -0.20, -0.06 | |
| Expressive language | -0.20* | -0.36, -0.04 | -0.02 | -0.16, 0.12 | 0.11 | -0.10, 0.31 | | 0.05 | -0.04, 0.13 | |
| London vs Manchester | -0.16 | -1.50, 1.18 | -0.95 | -1.72, -0.19 | 0.90 | -0.74, 2.53 | | -0.22 | -0.94, 0.50 | |
| London vs Newcastle | -0.31 | -1.80, 1.18 | 0.07 | -0.84, 0.99 | 0.18 | -1.33, 1.70 | | 0.08 | -0.72, 0.88 | |
| Sex | 1.95* | 0.05, 3.84 | 1.41* | 0.27, 2.54 | 0.60 | -1.44, 2.63 | | 0.85 | -0.16, 1.87 | |
| Age group | 0.94 | -0.25, 2.13 | 1.44** | 0.76, 2.11 | 0.79 | -0.54, 2.12 | | 0.63 | -0.01, 1.27 | |
| Parent’s job | -0.22 | -1.50, 1.06 | 0.04 | -0.67, 0.76 | -0.73 | -2.38, 0.92 | | -0.12 | -0.81, 0.56 | |
| Parent’s education | 0.05 | -1.37, 1.48 | 1.19* | 0.35, 2.03 | 1.43 | -0.46, 3.32 | | 0.85 | 0.08, 1.61 | |
| Effect size (95% CI) |  | | -0.24 (-0.64, 0.17) | | -0.01 (-0.75, 0.73) | | |  | | |
|  | -0.10 (-0.42, 0.23) | | -0.19 (-0.54, 0.17) | | | | | -0.25 (0.67, 0.16) | | |

*Note.* Negative effect sizes are in favour of PACT. Cognitive ability (nonverbal) is measured with the Mullen Scales for Early Learning. Expressive language is measured with the Preschool Language Scales. Effect sizes are calculated using the pooled standard deviation at baseline. ADOS = Autism Diagnostic Observation Schedule; ADOS-BOSCC = Brief Observation of Social Communication Change-version for ADOS; RRB = Restricted and Repetitive Behaviour; *n* = 104. p < .05 ** p < .001

**Table S12**

*Effect Sizes [95% CI] for Intervention Models using Change Score Variation*

|  | Overall (n = 146) | Module 1 (n =104) |
| --- | --- | --- |
| ADOS-BOSCC |  | -0.25 [-0.60, 0.09] |
| ADOS | Module 1 No words: -0.43 [-0.87, 0.009]  Module 1 Some words: -0.02 [-0.59, 0.55]  Module 2: -0.65 [-1.10, -0.19]  Overall: -0.41 [-0.69, -0.14] | Module 1 No words: -0.46* [-0.91, -0.01]  Module 1 Some words: 0.06 [-0.53, 0.64]  Overall: -0.27 [-0.70, 0.01] |
| ADOS CSS | -0.34* [-0.59. -0.10] | -0.20 [-0.52, 0.11] |

*Note.* ADOS = Autism Diagnostic Observation Schedule; ADOS-BOSCC = Brief Observation of Social and Communication Change-version for ADOS; * p<0.05

**Table S13**

*Pre-post Change Scores (SD) for ADOS-BOSCC and ADOS by Intervention Group for full PACT sample*

|  | Baseline | | Endpoint | | TAU change | | PACT change | |
| --- | --- | --- | --- | --- | --- | --- | --- | --- |
|  | Mean (SD) | | Mean (SD) | | Mean difference (SD) | Effect size (*d_z_*) | Mean difference (SD) | Effect size (*d_z_*) |
|  | TAU | PACT | TAU | PACT |  |  |  |  |
| ADOS Total  ADOS CSS | 19.9 (3.61)  7.90 (1.39) | 20.0 (4.16)  7.99 (1.39) | 18.3 (5.17)  7.28 (1.62) | 16.4 (5.65)  6.74 (1.74) | -1.57 (4.10)*  -0.63 (1.78)* | 0.38  0.47 | -3.54 (4.62)**  -1.24 (1.73)** | 0.77  0.86 |
| ADOS SA algorithm  ADOS CSS SA | 15.2 (3.00)  7.75 (1.39) | 15.1 (3.23)  7.80 (1.40) | 13.7 (4.41)  7.21 (1.91) | 12.4 (4.46)  6.66 (1.80) | -1.53 (3.61)**  -0.54 (2.01)* | 0.42  0.27 | -2.64 (4.16)**  -1.14 (1.97)** | 0.63  0.57 |
| ADOS RRB algorithm  ADOS CSS RRB | 4.68 (1.70)  7.93 (1.52) | 4.93 (1.93)  8.15 (1.52) | 4.64 (1.87)  7.88 (1.52) | 4.03 (1.97)  7.31 (2.13) | -0.04 (2.02)  -0.06 (1.80) | 0.02  0.03 | -0.91 (2.10)**  -0.84 (2.00)** | 0.43  0.42 |

*Note.*

A negative change score indicates improvem ent on the scale. Paired sample t-tests tested within-subdomain change. ADOS = Autism Diagnostic Observation Schedule, *d_z_ =* Cohen’s d_z_ effect size for correlated samples, PACT = Preschool Autism Communication Trial, SA = social affect, SC = social communication, RRB = restricted and repetitive behaviours, TAU = treatment as usual. ADOS algorithm and ADOS CSS (*n* = 146 [TAU n = 72; PACT n = 74]).

*p<.05, ** p<.001

**Figure S3**

Forest Plot of Intervention Effect Size Estimates for the ADOS Algorithm and ADOS CSS Total and Subdomain Scores for the Full PACT Sample. Negative effect sizes are in favour of PACT. ADOS = Autism Diagnostic Observation Schedule; PACT = Preschool Autism Communication Trial; RRB = restricted and repetitive behaviours; TAU = treatment as usual; *n* = 152

**Table S14**

*Intervention Effect Results for ADOS Algorithm and ADOS CSS for the Full PACT Sample*

|  | ADOS Algorithm Total | | | | | | ADOS CSS | | | |
| --- | --- | --- | --- | --- | --- | --- | --- | --- | --- | --- |
|  | Module 1 | | | | Module 2 | | Module 1 | | Module 2 | |
| Strata at baseline | Few to no words | | Some words | |  |  |  |  |  |  |
|  | Coefficient | 95% CI | Coefficient | 95% CI | Coefficient | 95% CI | Coefficient | 95% CI | Coefficient | 95% CI |
| PACT intervention | -2.00 | -4.04, 0.04 | -0.08 | -2.62, 2.46 | -2.64** | -4.50, -0.78 | -0.31 | -.87, .24 | -1.05* | -1.70, -0.39 |
| Covariates |  |  |  |  |  |  |  |  |  |  |
| Cognitive ability (nonverbal) | -0.33* | -0.62, -0.03 | -0.36* | -0.59, -0.14 | -0.25* | -0.40, -0.10 | -0.12** | -0.19, -0.06 | -0.08* | -0.13, -0.03 |
| Expressive language | -0.34 | -0.75, 0.06 | 0.33 | -0.13, 0.79 | 0.14 | -0.04, 0.32 | 0.005 | -0.07, 0.08 | 0.04 | -0.03, 0.10 |
| London vs Manchester | -4.18** | -6.32, -2.03 | -0.75 | -4.30, 2.80 | -5.63** | -8.55, -2.71 | -0.96* | -1.62, -0.31 | -1.62* | -2.62, -0.61 |
| London vs Newcastle | -1.60 | -4.19, 1.00 | -0.62 | -3.82, 2.58 | -2.03 | -4.69, 0.64 | -0.61 | -1.32, 0.11 | -0.71 | -1.62, 0.21 |
| Sex | 1.37 | -1.95, 4.69 | 0.36 | -4.22, 4.94 | 5.21* | 2.27, 8.15 | 0.53 | -0.42, 1.48 | 2.06** | 1.05, 3.07 |
| Age group | 1.63 | -0.28, 3.54 | -0.69 | -3.65, 2.26 | -1.62 | -5.11, 1.88 | -0.05 | -0.64, 0.53 | -0.49 | -1.69, 0.71 |
| Parent’s job | 1.15 | -0.92, 3.21 | -2.27 | -5.93, 1.39 | 0.17 | -2.19, 2.54 | 0.26 | -0.37, 0.89 | 0.10 | -0.71, 0.92 |
| Parent’s education | 1.04 | -1.37, 3.45 | 3.76 | -0.38, 7.89 | 3.07* | 0.66, 5.48 | 0.23 | -0.47, 0.93 | 1.26* | 0.43, 2.09 |
| Effect size (95% CI) | -0.67 (-1.36, 0.01) | | -0.03 (-0.96, 0.90) | | -0.72* (-1.22, -0.21) | | -0.22 (-0.62, 0.17) | | -.77 (-1.25, -0.29) | |
|  | -0.59* (-0.97, -0.22) | | | | | | -0.45 (-0.75, -0.14) | | | |

*Note.* Negative effect sizes are in favour of PACT. Cognitive ability (nonverbal) is measured with the Mullen Scales for Early Learning. Expressive language is measured with the Preschool Language Scales. Effect sizes are calculated using the pooled standard deviation at baseline. ADOS = Autism Diagnostic Observation Schedule; ADOS-BOSCC = Brief Observation of Social Communication Change-version for ADOS; RRB = Restricted and Repetitive Behaviour; *n* = 152. * p < .05 ** p < .001

**Table S15**

*Intervention Effect Results for ADOS Algorithm and ADOS CSS Social Affect subdomains for the Full PACT Sample*

|  | ADOS Algorithm SA | | | | | | ADOS CSS SA | | | |
| --- | --- | --- | --- | --- | --- | --- | --- | --- | --- | --- |
|  | Module 1 | | | | Module 2 | | Module 1 | | Module 2 | |
|  | Few to no words | | Some words | |  |  |  |  |  |  |
|  | Coefficient | 95% CI | Coefficient | 95% CI | Coefficient | 95% CI | Coefficient | 95% CI | Coefficient | 95% CI |
| PACT intervention | -1.28 | -3.01, 0.44 | -0.21 | -2.45, 2.03 | -1.09 | -2.68, 0.50 | -0.43* | -1.06, 0.20 | -0.41 | -1.25, 0.43 |
| Covariates |  |  |  |  |  |  |  |  |  |  |
| Cognitive ability (nonverbal) | -0.13 | -0.38, 0.13 | -0.26* | -0.45, -0.06 | -0.16* | -0.29, -0.03 | -0.11* | -0.18, -0.03 | -0.10* | -0.17, -0.03 |
| Expressive language | -0.34* | -0.69, -0.001 | 0.21 | -0.18, 0.61 | 0.15 | -.006, 0.30 | 0.06 | -0.04, 0.15 | 0.05 | -0.03, 0.13 |
| London vs Manchester | -3.26** | -5.08, -1.43 | -1.17 | -4.25, 1.92 | -6.44** | -8.90, -3.97 | -1.10* | -1.85, -0.36 | -2.99** | -4.30, -1.67 |
| London vs Newcastle | -1.71 | -3.92, 0.50 | -0.54 | -3.32, 2.24 | -2.21 | -4.46, 0.04 | -0.81 | -1.63, 0.005 | -0.91 | -2.11, 0.29 |
| Sex | -0.14 | -2.97, 2.69 | -0.02 | -3.99, 3.95 | 4.45** | 1.96, 6.93 | -0.16 | -1.25, 0.93 | 2.44** | 1.12, 3.77 |
| Age group | 0.22 | -1.41, 1.86 | -1.41 | -3.98, 1.17 | -0.73 | -3.68, 2.22 | -0.50 | -1.17, 0.17 | -0.04 | -1.61, 1.53 |
| Parent’s job | 1.02 | -0.74, 2.78 | -1.43 | -4.61, 1.76 | 0.57 | -1.42, 2.57 | 0.16 | -0.56, 0.88 | 0.27 | -0.79, 1.34 |
| Parent’s education | -0.25 | -2.30, 1.79 | 2.61 | -0.97, 6.20 | 2.18 | 0.14, 4.21 | -0.03 | -0.83, 0.77 | 1.19* | 0.10, 2.28 |
| Effect size (95% CI) | -0.60 (-1.40, 0.21) | | -0.09 (-1.01, 0.84) | | -0.37 (-0.92, 0.17) | | -0.31 (-0.77, 0.15) | | -0.31 (-0.94, 0.32) | |
|  | -0.38* (-0.78, -0.03) | | | | | | -0.31 (-0.68, 0.06) | | | |

*Note.* Negative effect sizes are in favour of PACT. Cognitive ability (nonverbal) is measured with the Mullen Scales for Early Learning. Expressive language is measured with the Preschool Language Scales. Effect sizes are calculated using the pooled standard deviation at baseline. ADOS = Autism Diagnostic Observation Schedule; ADOS-BOSCC = Brief Observation of Social Communication Change-version for ADOS; RRB = Restricted and Repetitive Behaviour; *n* = 152.* p < .05 ** p < .001

**Table S16**

*Intervention Effect Results for ADOS Algorithm and ADOS CSS RRB subdomains for the Full PACT Sample*

|  | ADOS Algorithm Total | | | | | | | | | | | ADOS CSS | | | | ADOS CSS | | |
| --- | --- | --- | --- | --- | --- | --- | --- | --- | --- | --- | --- | --- | --- | --- | --- | --- | --- | --- |
|  | Module 1 | | | | | | | Module 2 | | | | Module 1 | | | | Module 2 | | |
| Strata at baseline | Few to no words | | | | Some words | | |  | |  | |  | |  | |  |  | |
|  | Coefficient | 95% CI | | Coefficient | | 95% CI | Coefficient | | 95% CI | | Coefficient | | 95% CI | | Coefficient | | | 95% CI |
| PACT intervention | -0.36 | -1.15, 0.43 | | -0.04 | | -1.08, 1.01 | -1.69** | | -2.44, -0.94 | | -0.29 | | -0.90, 0.33 | | -1.97** | | | -2.88, -1.06 |
| Covariates |  |  | |  | |  |  | |  | |  | |  | |  | | |  |
| Cognitive ability (nonverbal) | -0.20** | -0.31, -0.09 | | -0.10* | | -0.20, -0.001 | -0.09* | | -0.15, -0.02 | | -0.11* | | -0.18, -0.04 | | -0.10* | | | -0.18, -0.02 |
| Expressive language | -0.01 | -0.16, 0.14 | | 0.11 | | -0.09, 0.31 | -0.006 | | -0.08, 0.07 | | 0.03 | | -0.05, 0.12 | | -0.02 | | | -0.12, 0.08 |
| London vs Manchester | -0.94* | -1.73, -0.15 | | 0.40 | | -1.20, 2.00 | 0.79 | | -0.49, 2.07 | | -0.36 | | -1.08, 0.36 | | 0.36 | | | -1.23, 1.95 |
| London vs Newcastle | 0.18 | -0.78, 1.14 | | -0.06 | | -1.50, 1.37 | 0.17 | | -0.99, 1.34 | | 0.003 | | -0.78, 0.79 | | -0.20 | | | -1.65, 1.25 |
| Sex | 1.49* | 0.27, 2.71 | | 0.42 | | -1.63, 2.48 | 0.75 | | -0.54, 2.03 | | 0.87 | | -0.18, 1.91 | | 0.35 | | | -1.26, 1.95 |
| Age group | 1.38** | 0.68, 2.09 | | 0.69 | | -0.64, 2.02 | -0.85 | | -2.37, 0.68 | | 0.61 | | -0.03, 1.25 | | -0.73 | | | -2.62, 1.17 |
| Parent’s job | 0.06 | -0.70, 0.82 | | -0.85 | | -2.50, 0.79 | -0.38 | | -1.41, 0.66 | | -0.06 | | -0.75, 0.64 | | 0.01 | | | -1.27, 1.30 |
| Parent’s education | 1.10* | 0.21, 1.99 | | 1.15 | | -0.71, 3.00 | 0.92 | | -0.13, 1.97 | | 0.81 | | 0.04, 1.58 | | 0.66 | | | -0.65, 1.97 |
| Effect size (95% CI) | -0.20 (-0.64, 0.24) | | | | -0.03 (-0.72, 0.67) | | | -0.86 (-1.24, -0.48) | | | | -0.19 (-0.60, 0.22) | | | | -1.24 (-1.81, -0.67) | | |
|  |  | |  | | -0.49* (-0.76, -0.23) | | | | | | | -0.54* (-0.87, -0.21) | | | | | | |

*Note.* Negative effect sizes are in favour of PACT. Cognitive ability (nonverbal) is measured with the Mullen Scales for Early Learning. Expressive language is measured with the Preschool Language Scales. Effect sizes are calculated using the pooled standard deviation at baseline. ADOS = Autism Diagnostic Observation Schedule; ADOS-BOSCC = Brief Observation of Social Communication Change-version for ADOS; RRB = Restricted and Repetitive Behaviour; *n* = 152. * p < .05 ** p < .001

Estimate (adjusted CI)

Effect size

**Figure S4**

Forest Plot of Intervention ‘Signature of Change’: Effect Estimates for the Items of the ADOS (Module 2) with 95% Confidence Intervals Corrected for Multiple Comparisons within each Measure with Effect Sizes (right hand side). This graph plots model estimates, whilst effect sizes are listed in the right-hand column. Confidence intervals of the model estimates were corrected using the Dubey/Armitage-Parmar adjustment which accounts for there being multiple correlated outcomes. ADOS = Autism Diagnostic Observation Schedule; JA = joint attention; M2 = Module 2; *n* = 35

**References**

Anagnostou, E., Jones, N., Huerta, M., Halladay, A. K., Wang, P., Scahill, L., . . . Dawson, G. (2015). Measuring social communication behaviors as a treatment endpoint in individuals with autism spectrum disorder. *Autism, 19*(5), 622-636. doi:10.1177/1362361314542955

de Bildt, A., Kraijer, D., Sytema, S., & Minderaa, R. (2005). The psychometric properties of the Vineland Adaptive Behavior Scales in children and adolescents with mental retardation. *Journal of Autism and Developmental Disorders, 35*(1), 53-62.

Green, J., Charman, T., McConachie, H., Aldred, C., Slonims, V., Howlin, P., . . . Pickles, A. (2010). Parent-mediated communication-focused treatment in children with autism (PACT): a randomised controlled trial. *The Lancet, 375*(9732), 2152-2160. doi:10.1016/s0140-6736(10)60587-9

Grzadzinski, R., & Lord, C. (2018). Commentary: Insights into the Development of the Brief Observation of Social Communication Change (BOSCC). *Journal of mental health & clinical psychology, 2*(5), 15.

Mullen, E. M. (1995). *Mullen scales of early learning*: AGS Circle Pines, MN.

Perry, A., & Factor, D. C. (1989). Psychometric validity and clinical usefulness of the Vineland Adaptive Behavior Scales and the AAMD Adaptive Behavior Scale for an autistic sample. *Journal of Autism and Developmental Disorders, 19*(1), 41-55.

Sparrow, S. S., Cicchetti, D. V., & Balla, D. A. (2005). *Vineland Adaptive Behavior Scales Vineland-II: Survey Forms Manual*: Pearson Minneapolis, MN.

Swineford, L. B., Guthrie, W., & Thurm, A. (2015). Convergent and divergent validity of the Mullen Scales of Early Learning in young children with and without autism spectrum disorder. *Psychological Assessment, 27*(4), 1364.

Zimmerman, I. L., Steiner, V. G., Pond, R. E., Boucher, J., & Lewis, V. (1997). *Preschool Language Scale-3 (UK)*: Psychological Corporation.
